# Crystal structures of *Lymphocytic choriomeningitis virus* endonuclease domain complexed with diketo-acid ligands

**DOI:** 10.1107/S2052252518001021

**Published:** 2018-02-22

**Authors:** Magali Saez-Ayala, Elsie Laban Yekwa, Mauro Carcelli, Bruno Canard, Karine Alvarez, François Ferron

**Affiliations:** a Aix-Marseille Université, AFMB UMR 7257, 13288 Marseille, France; b CNRS, AFMB UMR 7257, 13288 Marseille, France; c Dipartimento di Scienze Chimiche, della Vita, della Sostenibilità Ambientale, Università Di Parma, Parco Area delle Scienze 17/A, 43124 Parma, Italy

**Keywords:** *Arenaviridae*, endonucleases, *Lymphocytic choriomeningitis virus*, LCMV, diketo acids, compound optimization, metal chelation

## Abstract

The *Arenaviridae* family, together with the *Bunyaviridae* and *Orthomyxoviridae* families, is one of the three negative-stranded RNA viral families that encode an endonuclease in their genome. The endonuclease domain is at the N-terminus of the L protein, a multifunctional protein that includes the RNA-dependent RNA polymerase. The synthesis of mRNA in arenaviruses is a process that is primed by capped nucleotides that are ‘stolen’ from the cellular mRNA by the endonuclease domain in cooperation with other domains of the L protein. This molecular mechanism has been demonstrated previously for the endonuclease of the prototype *Lymphocytic choriomeningitis virus* (LCMV). However, the mode of action of this enzyme is not fully understood as the original structure did not contain catalytic metal ions. The pivotal role played by the cap-snatching process in the life cycle of the virus and the highly conserved nature of the endonuclease domain make it a target of choice for the development of novel antiviral therapies. Here, the binding affinities of two diketo-acid (DKA) compounds (DPBA and L-742,001) for the endonuclease domain of LCMV were evaluated using biophysical methods. X-ray structures of the LCMV endonuclease domain with catalytic ions in complex with these two compounds were determined, and their efficacies were assessed in an *in vitro* endonuclease-activity assay. Based on these data and computational simulation, two new DKAs were synthesized. The LCMV endonuclease domain exhibits a good affinity for these DKAs, making them a good starting point for the design of arenavirus endonuclease inhibitors. In addition to providing the first example of an X-ray structure of an arenavirus endonuclease incorporating a ligand, this study provides a proof of concept that the design of optimized inhibitors against the arenavirus endonuclease is possible.

## Abstract 2

Crystal structures of the endonuclease domain of *Lymphocytic choriomeningitis virus* (LCMV) in complex with two diketo-acid (DKA) compounds were determined. Based on these data and activity assays, two new DKAs with good affinity for the LCMV endonuclease domain were synthesized.

## 1. Introduction   

The *Arenaviridae* is a family of viruses that are associated with rodent-transmitted infections in humans. These viruses cause chronic and asymptomatic infections in rodents, and constitute a reservoir of human pathogens across the world (Buchmeier *et al.*, 2007 ▸). Indeed, several arenaviruses, including *Lassa virus*, *Lujo virus*, *Guanarito virus*, *Sabiá virus*, *Chapare virus*, *Machupo virus* and *Junín virus*, are responsible for outbreaks of haemorrhagic fever and constitute a major public health concern (Bowen *et al.*, 1997 ▸; Enria *et al.*, 2008 ▸; Briese *et al.*, 2009 ▸). In West Africa alone (Sierra Leone, Guinea, Liberia, Nigeria and Benin), *Lassa virus* is responsible for several hundred thousand infections per year (Günther & Lenz, 2004 ▸). It is a common endemic infection that in most cases leads to hearing loss, tremors and encephalitis, and in 1% of cases becomes a deadly haemorrhagic fever (Yun *et al.*, 2015 ▸). The last Lassa fever outbreak, in Nigeria between August 2015 and May 2016, had a case-fatality rate (CFR) of 53.9% based on confirmed WHO data of 165 cases and 89 deaths (confirmed through laboratory testing; World Health Organization, 2016 ▸). *Lymphocytic chorio­meningitis virus* (LCMV) was the first arenavirus to be isolated and is considered to be the prototype virus for the family. Responsible for occasional transmission to man, it may result in life-threatening meningitis and/or haemorrhagic fever, and several clinical studies have suggested that its pathogenicity has been underestimated (Mets *et al.*, 2000 ▸; Schulte *et al.*, 2006 ▸; Jamieson *et al.*, 2006 ▸; Bonthius, 2012 ▸). Because its natural host is the common house mouse (*Mus musculus*), it has a global distribution, with LCMV infections reported in Europe, the Americas, Australia and Japan. LCMV is a human pathogen of significant clinical relevance, causing central nervous system disease, congenital malformation, choriomeningitis, and systemic and highly fatal infection in immunocompromised organ-transplant recipient patients (Fisher-Hoch *et al.*, 1995 ▸; Mets *et al.*, 2000 ▸; Barton *et al.*, 2002 ▸; Fischer *et al.*, 2006 ▸; Schulte *et al.*, 2006 ▸; MacNeil *et al.*, 2012 ▸). Humans are generally infected by arenaviruses through exposure to aerosols of fresh urine, droppings, saliva or nesting materials from infected rodents or during organ transplantation (Fischer *et al.*, 2006 ▸). Despite the widespread threat to human health that these pathogens represent, there are no vaccines and only limited therapeutic options (Lee *et al.*, 2011 ▸). The only licensed drug for the treatment of human arenavirus infection is the broad-spectrum antiviral ribavirin, which is only partially effective and is associated with significant toxicity (McCormick *et al.*, 1986 ▸; Kilgore *et al.*, 1997 ▸; Enria *et al.*, 2008 ▸). Therefore, there is an unmet need to identify novel compounds that could be developed into antiviral drugs to combat human-pathogenic arenaviruses.

Arenaviruses are enveloped viruses with a bi-segmented negative single-stranded RNA genome. Each segment encodes two proteins separated by an intergenic region (IGR) in an ambisense manner. The L RNA segment encodes the large protein L (∼200 kDa) and the small disordered protein Z (∼11 kDa) (Buchmeier *et al.*, 2007 ▸). L (UniProt P14240) is a multi-domain protein: its N-terminus includes an endo­nuclease domain (residues 1–196) followed by a viral RNA-dependent RNA polymerase (RdRp) domain, but it lacks a capping machinery (López *et al.*, 2001 ▸). The Z protein, which contains a RING finger motif, is a multifunction protein that regulates the life cycle of the virus and, during budding, assembles to form the matrix (Cornu & de la Torre, 2001 ▸; Perez *et al.*, 2003 ▸, 2004 ▸; Hastie, Zandonatti *et al.*, 2016 ▸). The S RNA segment encodes the precursor of the mature virion glycoprotein GP-C (75 kDa), which after post-translational cleavage gives GP-1 (40–46 kDa) and GP-2 (35 kDa) (Hastie, Igonet *et al.*, 2016 ▸), and the nucleoprotein NP (∼63 kDa; Buchmeier *et al.*, 2007 ▸). NP forms a polymer that protects the genomic (and antigenomic) RNA (RNA_v_). L and NP together with RNA_v_ form an active ribonucleic complex for replication and transcription (Pinschewer *et al.*, 2003 ▸). Arenaviruses belongs to the negative RNA viruses; therefore, the first step in the viral cycle starts by the transcription of L and NP viral messenger RNAs (mRNAs; Perez & de la Torre, 2003 ▸). This step is undertaken by the L protein and starts (possibly with the help of NP) by recruiting cellular mRNA as a first step. The L protein cleaves and snatches the native cellular mRNA cap structure through its endonuclease domain after ∼11 nucleotides, and uses it as a primer sequence for the RdRp domain of the L protein to synthesize the viral mRNA. The crystal structure of the LCMV endonuclease domain (ENDO) was originally determined some time ago (Morin *et al.*, 2010 ▸), but in this original structure the catalytic ions necessary to fully understand the mode of action of the endonuclease were missing. Cap-snatching is essential to the life cycle of the virus, and this makes the endonuclease domain a target of choice for drug-design development (Charrel *et al.*, 2011 ▸). This mechanism is shared by *Orthomyxoviridae* and *Bunyaviridae*, two families that harbour prominent pathogenic members and the endonucleases of which have been used in drug development (Reguera *et al.*, 2010 ▸; Kowalinski *et al.*, 2012 ▸; DuBois *et al.*, 2012 ▸). However, *Arenaviridae* endonuclease is a challenging target for inhibitor development. Indeed, it has the most phylogenetically remote structure compared with the enzymes from *Orthomyxoviridae* and *Bunyaviridae* (Ferron *et al.*, 2017 ▸). Previous structural studies of *Lassa virus* endo­nuclease characterized the complex with catalytic ions (Wallat *et al.*, 2014 ▸), but failed to obtain a complex with an inhibitor (Reguera *et al.*, 2016 ▸). Nevertheless, there is great interest in viral endonuclease inhibitors that are optimally designed to occupy the active site and chelate the metal ions (Rogolino *et al.*, 2012 ▸).

**Figure 1:**
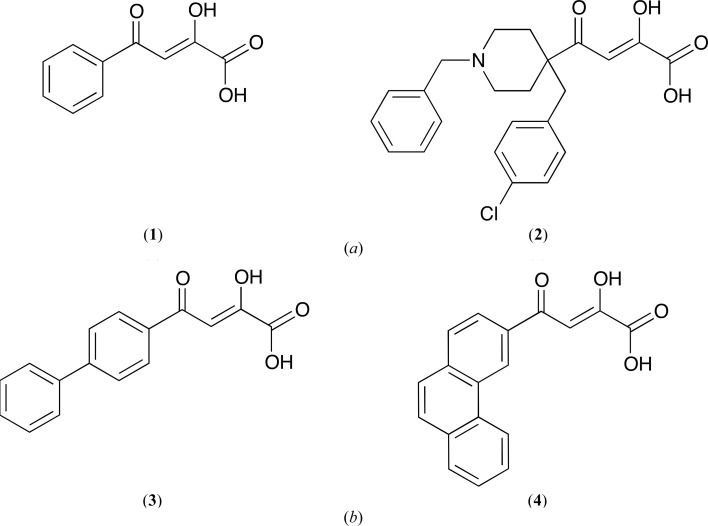
Structures of 2,4-dioxo-4-phenylbutanoic acid [DPBA, (**1**)], (*Z*)-4-{1-benzyl-4-[(4-chlorophenyl)­methyl]­piperidin-4-yl}-2-hydroxy-4-oxobut-2-enoic acid [L-742,001, (**2**)], 2-hydroxy-4-(biphenyl-4-yl)-4-oxobut-2-enoic acid (**3**) and 2-hydroxy-4-oxo-4-(phenanthren-3-yl)but-2-enoic acid (**4**).

In this study, we selected two molecules that are known to target the influenza virus endonuclease (*Orthomyxoviridae*) as a starting point for the structure-based drug design of anti-arenavirus compounds (Noble *et al.*, 2012 ▸; Stevaert *et al.*, 2013 ▸). We characterized the binding affinity of DPBA (**1**) and L-742,001 (**2**) (Fig. 1 ▸ *a*) for the LCMV ENDO by biophysical methods. We evaluated their efficacy in an *in vitro* endo­nuclease assay and solved crystal structures of LCMV ENDO with catalytic ions (Mg^2+^ or Mn^2+^) and each of these two diketo acids (DKAs). Based on the structure analysis and *in silico* analysis, we synthesized two new DKAs, compounds (**3**) and (**4**) (Fig. 1 ▸ *b*), which exhibited not only a better affinity for LCMV ENDO but also a better inhibition of its activity.

## 2. Materials and methods   

### 2.1. Sequence analysis   

All available full-length sequences of *Arenaviridae* L proteins were downloaded from NCBI. Using the redundancy option in *Jalview*, all identical sequences were removed. The working set was composed of 384 sequences, including *Mammarenavirus* and *Reptiarenavirus* sequences. This subset was aligned with *MUSCLE* (Edgar, 2004 ▸) using the balanced option optimized for long sequences and large data sets. The first 220 amino acids corresponding to an extended endo­nuclease domain were selected. The working set was again purged of identical sequences, leaving 245 sequences. The resulting alignment was analysed to specifically target identical residues and the information was correlated with the LCMV endonuclease structure. The motif conservation was represented with *WebLogo* (Crooks *et al.*, 2004 ▸). A list of the sequences used is summarized in Supplementary Table S1 and an alignment performed with *ESPript* (Gouet *et al.*, 2003 ▸) is shown in Supplementary Fig. S1.

### 2.2. Protein expression and purification   

Wild-type ENDO (ENDO-WT) and its D118A and D88A mutants were cloned into pDEST14 with an N-terminal hexahistidine tag and expressed in *Escherichia coli* Rosetta (DE3) pLysS cells at 17°C in TB medium overnight after induction with 500 m*M* IPTG. Cell pellets from harvested cultures were resuspended in 50 m*M* Tris buffer pH 8.0, 300 m*M* NaCl, 10 m*M* imidazole, 0.1% Triton X-100, 5% glycerol. Lysozyme (0.25 mg ml^−1^), DNase I (10 µg ml^−1^) and EDTA-free protease-inhibitor cocktail (Roche) were added before sonication. Immobilized metal-ion chromatography of the clarified lysates was performed on a 5 ml HisPrep column (ÄKTA­xpress FPLC system, GE Healthcare) eluted using the same buffer with 500 m*M* imidazole. The eluted His-tagged fraction was diluted and purified on a HiTrap Q Sepharose 1 ml column (GE Healthcare). Proteins were eluted using a linear gradient from 50 m*M* to 1 *M* NaCl in 10 m*M* HEPES buffer pH 7.5, 2 m*M* DTT. Size-exclusion chromatography was performed on a preparative Superdex 200 column (GE Healthcare) pre-equilibrated with 10 m*M* HEPES pH 8.0, 50 m*M* NaCl, 2 m*M* DTT. The protein was concentrated to 25 mg ml^−1^ and frozen in liquid nitrogen.

### 2.3. Compounds and substrate characterization   

#### 2.3.1. Differential scanning fluorimetry (DSF)   

Melting-temperature (*T*_m_) values of the proteins were determined by a thermofluorescence-based assay. In 96-well thin-walled PCR plates, 11 µl protein solution (ENDO) was added to 11 µl of compound (**1**) or compound (**2**) and/or metal ions (Mg^2+^ or Mn^2+^) in 10 m*M* HEPES buffer pH 8.0, 50 m*M* NaCl, 2 m*M* DTT. Finally, 3 µl of the fluorescent dye SYPRO Orange was added (715-fold diluted in H_2_O; Molecular Probes). The thermal denaturation of the proteins was followed by measuring the fluorescence emission at 575 nm (with excitation at 490 nm) using a CFX Connect Real-Time PCR Detection System (Bio-Rad). The final concentrations were adjusted to 75 µ*M* protein, 0.5 m*M* MgCl_2_, 0.5 m*M* MnCl_2_, 450 µ*M* compound (**1**) or (**2**) (final ligand:protein ratio = 6) and 5% DMSO. The denaturation midpoints of the proteins were calculated with the Boltzmann equation using *GraphPad Prism*. All measurements were performed in triplicate.

#### 2.3.2. Microscale thermophoresis (MST)   

MST experiments were performed on a Monolith NT.115 instrument (NanoTemper Technologies). Proteins were labelled with the red fluorescent dye NT-647 using the Protein Labeling Kit RED-NHS (NanoTemper Technologies). The concentration of the labelled protein was kept constant at 100 n*M*, while the concentration of the compound was varied. A 15-step twofold dilution series beginning at 500, 250 or 125 µ*M* finally yielded 16 different concentrations of the tested compound [(**1**)–(**4**)]. Experiments were carried out in 10 m*M* HEPES buffer pH 8 containing 100 m*M* NaCl, 1 m*M* DTT, 0.05%(*w*/*v*) Tween 20, 0.25 m*M* MgCl_2_ and 0.25 m*M* MnCl_2_. The final samples were adjusted to 5% DMSO to ensure the solubility of the compounds. The samples were centrifuged for 5 min at 13 000 rev min^−1^ to remove potential aggregates and the supernatant was loaded into standard treated MST-grade glass capillaries (NanoTemper Technologies). After a 5 min incubation period the MST was measured with 80% LED power and 80% infra-red laser power. *K*_d_ values were determined using the *NanoTemper* analysis software.

#### 2.3.3. Isothermal titration calorimetry (ITC)   

Purified ENDO was diluted in ITC buffer consisting of 10 m*M* HEPES pH 8.0, 50 m*M* NaCl, 2 m*M* DTT, 0.25 m*M* MgCl_2_, 0.25 m*M* MnCl_2_. Compound (**1**) was diluted in the same buffer. The final DMSO concentration was 5% in the cell and in the syringe. Titrations were carried out in a MicroCal ITC200 microcalorimeter (GE Healthcare). Experiments were designed with a titrant concentration [1 m*M* DPBA (**1**) in the syringe] ten times the analyte concentration (100 µ*M* protein in the cell), using 19 injections at 25°C. A first small injection (0.2 µl) was included in the titration protocol in order to remove air bubbles trapped in the syringe prior to titration. Raw data were scaled after setting the zero to the titration saturation heat value. Integrated raw ITC data were fitted to a one-site nonlinear least-squares fit model using the *MicroCal Origin* plugin as implemented in *Origin* 9.1 (OriginLab). Each experiment was performed three times and the data are presented as the mean ± standard deviation.

#### 2.3.4. *In vitro* endonuclease assay   

An *in vitro* endo­nuclease assay was performed to investigate the inhibition of compounds (**1**) and (**2**). The reaction was carried out in 20 µl samples consisting of 20 m*M* Tris–HCl pH 8, 150 m*M* NaCl, 1 m*M* TCEP, 2 m*M* MnCl_2_. ENDO-WT (20 µ*M*) was premixed with 50, 125, 250, 500, 1000 or 2000 µ*M* of each compound and the reaction was started by adding 1 µ*M* of a 19-nucleotide single-stranded RNA (Morin *et al.*, 2010 ▸). The reaction mixtures were incubated at 25°C and the reaction was stopped after 6 h by adding 20 µl loading buffer (formamide containing 10 m*M* EDTA). For experiments to compare the inhibition of DPBA (**1**) and compounds (**3**) and (**4**), ENDO-WT (20 µ*M*) was premixed with 50 µ*M* of each compound and the same protocol as above was applied. The reaction products were analyzed on 20% polyacrylamide/8 *M* urea gels. The un­digested RNAs were visualized on photostimulated plates using a Fluorescent Image Analyzer FLA3000 (Fuji) and quantified using *Image Gauge* (Fuji). Graphs were plotted using *GraphPad Prism*. All experiments were performed in triplicate.

### 2.4. Crystallization, data collection and structure determination   

#### 2.4.1. Crystallization   

All crystals were grown at 20°C using the sitting-drop vapour-diffusion method in 96-well plates (Greiner) by mixing 200 nl protein solution (13 mg ml^−1^ in 10 m*M* HEPES pH 8.0, 50 m*M* NaCl, 2 m*M* DTT) with 100 nl reservoir solution using a Mosquito robot (TTP Labtech). Crystals of ENDO-WT complexed with ions grew in a reservoir solution consisting of 100 m*M* sodium citrate pH 6.2, 3% isopropanol using protein solution supplemented with 1 m*M* MgCl_2_. Crystals of ENDO-WT complexed with ions and DPBA (**1**) grew in a reservoir solution consisting of 100 m*M* sodium citrate pH 6.2, 3.5% isopropanol using protein solution supplemented with 1 m*M* MgCl_2_ and 2 m*M* DPBA (**1**) (solubilized in PEG 400). Crystals of ENDO-WT complexed with ions and L-742,001 (**2**) grew in a reservoir solution consisting of 100 m*M* sodium citrate pH 6, 3.5% 2-propanol using protein solution supplemented with 1 m*M* MnCl_2_ and 2 m*M* L-742,001 (**2**) (solubilized in PEG 400). Crystals of the ENDO-D118A mutant grew in a reservoir solution consisting of 100 m*M* sodium citrate pH 6.3, 8% 2-propanol. All crystals were cryocooled in liquid N_2_ using 20%(*v*/*v*) glycerol in mother liquor as a cryoprotectant.

#### 2.4.2. Data collection and structure determination of LCMV   

The data for ENDO-WT complexed with ions were collected on beamline ID23-1 and the data for ENDO-WT complexed with L-742,001 (**2**) were collected on beamline ID23-2 at the European Synchrotron Radiation Facility (ESRF). The data for ENDO-WT complexed with DPBA (**1**) and the ENDO-D118A mutant were collected on the PROXIMA-1 beamline at Synchrotron SOLEIL. Data were processed, analysed and scaled using the *autoPROC* toolbox (Vonrhein *et al.*, 2011 ▸). Phases were obtained by molecular replacement using *Phaser* (McCoy *et al.*, 2007 ▸) with PDB entry 3jsb (Morin *et al.*, 2010 ▸) as a search model. All water and ligand molecules were removed from the search structure. Rebuilding of the initial model using *AutoBuild* (Terwilliger *et al.*, 2008 ▸) was then performed. Subsequently, all-atom isotropic temperature-factor refinement cycles were performed with *phenix.refine* (Afonine *et al.*, 2012 ▸) and/or with *BUSTER* (Blanc *et al.*, 2004 ▸). Electron-density maps were inspected using *Coot* (Emsley *et al.*, 2010 ▸). Extra density accounting for ions and/or compounds was observed for the complexed structures, but none was found for the mutant structures (Supplementary Fig. S4*b*). The structures were evaluated using *MolProbity* (Chen *et al.*, 2010 ▸) and *PROCHECK* (Laskowski *et al.*, 1993 ▸). Structural analysis was performed and high-resolution figures were produced with *UCSF Chimera* (Pettersen *et al.*, 2004 ▸). Data-collection and refinement statistics are given in Table 1 ▸.

### 2.5. Molecular docking   

For the molecular docking, we used the crystal structures of ENDO-WT complexes with different ions. The three-dimensional structure of ENDO-WT was energy-minimized by the steepest-gradient method of energy minimization followed by conjugate-gradient minimization, using the *MMTK* and *Amber* packages (Cornell *et al.*, 1995 ▸; Hinsen, 2000 ▸; Lindorff-Larsen *et al.*, 2010 ▸). Compounds were designed using *BKChem* v.0.13.0 (Kosata & Danne, 2010 ▸) and geometry-restrained using *eLBOW* (Moriarty *et al.*, 2009 ▸). The Mol2 and PDB file formats of the ligands and receptor were converted to PDBQT format using *UCSF Chimera* prior to docking. All of the water and solvent atoms of the protein were removed and the polar hydrogens and polar charge were added to the ions and ligand prior to docking. The protein was kept rigid while the ligand was allowed to rotate and explore more flexible binding pockets. The docking of the ligands onto ENDO-WT was performed iteratively using *AutoDock Vina* v.1.1.2 (Trott & Olson, 2010 ▸). The best poses from the first round of docking were used as seeds for the second round. The grid box size was initially 40 × 40 × 40 in order to verify that our ligands will preferentially bind in the catalytic site. The grid box size was further optimized to 23.2 × 15.6 × 21.2, thus covering the binding pockets; the default scoring function was used for docking.

Binding modes of the docked complexes were obtained and sorted based on their binding energy; ions and amino-acid residues that were present at a distance less of 3 Å were considered to be binding partners of the ligands. The binding modes were compared with these of the native structure. Figures showing the interactions of the docked complexes were generated using *UCSF Chimera* (Pettersen *et al.*, 2004 ▸).

### 2.6. Chemicals and chemistry   

2,4-Dioxo-4-phenylbutanoic acid [DPBA, (**1**)] was purchased from Interchim. (*Z*)-4-[1-Benzyl-4-[(4-chlorophenyl)­methyl]­piperidin-4-yl]-2-hydroxy-4-oxobut-2-enoic acid [L-742,001, (**2**)] was synthesized as previously reported (Stevaert *et al.*, 2015 ▸). 2-Hydroxy-4-(biphenyl-4-yl)-4-oxobut-2-enoic acid (**3**) and 2-hydroxy-4-oxo-4-(phenanthren-3-yl)but-2-enoic acid (**4**) were synthesized as described in the literature (Patil *et al.*, 2007 ▸; Bhatt *et al.*, 2011 ▸) and the conditions used for their preparation are detailed in Appendix *A* [App appa].

## 3. Results and discussion   

The endonuclease catalytic reaction is driven by two-metal-ion catalysis (TMIC): (i) to form a stable intermediate and then (ii) to break the phosphodiester backbone of the nucleic acid chain (Palermo *et al.*, 2015 ▸). Indeed, as shown by Steitz & Steitz (1993 ▸), the TMIC reaction is driven by two divalent metal ions positioned on each side of the targeted scissile phosphate bond to be cleaved. The metal ions facilitate an S_N_2-type reaction, in which one metal ion favours the formation of the nucleophilic O atom from a water molecule and the other helps the exit of the leaving group by associating with a nonbridging phosphoryl O atom. The catalytic centre of the ENDO resides in the N-terminal part around Asp88 and should contain two Mg^2+^ (or Mn^2+^) ions, which are critical for the RNA-cleavage mechanism. Our strategy to design inhibitors of this activity is to develop compounds that are able to block and inhibit the catalytic centre through the trapping of critical metal ions, resulting in functional impairment.

DPBA (**1**) and L-742,001 (**2**) are DKAs that carry a chelating motif consisting of a γ-ketone, an enolizable α-ketone and a carboxylic acid that is able to chelate the divalent metal ions that are present in the active sites of some enzymes (Kowalinski *et al.*, 2012 ▸; DuBois *et al.*, 2012 ▸; Fig. 1 ▸). These compounds have been identified as potent inhibitors of the activity of influenza PA N_ter_ in enzymatic assays or cell cultures and mouse models (Reguera *et al.*, 2010 ▸; Carcelli *et al.*, 2014 ▸; Stevaert *et al.*, 2015 ▸). This prompted us to investigate whether they can also have a similar effect on LCMV ENDO, which is a homologue of influenza PA N_ter_.

### 3.1. Biophysical characterization of the effect of DKA compounds on ENDO   

#### 3.1.1. Thermal stability by DSF   

**Figure 2:**
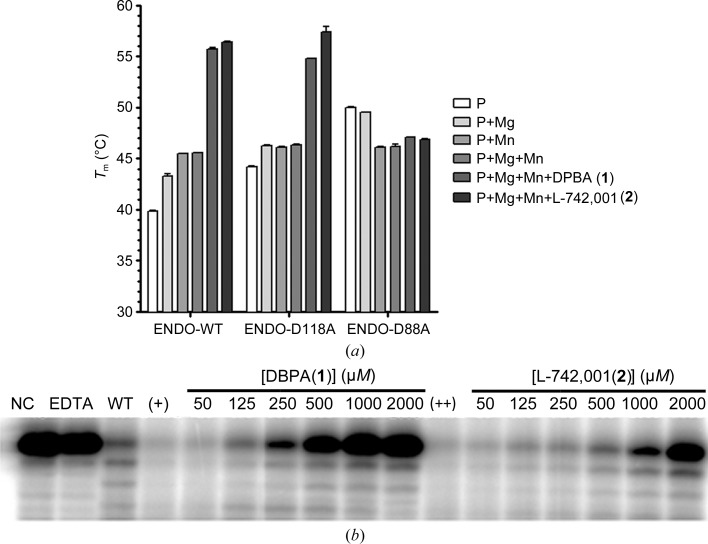
(*a*) Thermal stability of ENDO proteins determined by differential scanning fluorimetry (DSF). The melting temperatures (*T*_m_) of ENDO-WT, ENDO-D118A and ENDO-D88A (75 µ*M*; P) with or without the indicated divalent cation (at 0.5 m*M*; P+Mg, P+Mn, P+Mg+Mn) and the compounds DPBA (**1**) (P+Mg+Mn+DPBA) and L-742,001 (**2**) (P+Mg+Mn+L-742,001) (at 450 µ*M*, ligand:protein ratio = 6) were measured in a thermofluorescence experiment. (*b*) Polyacrylamide/8 *M* urea gels of inhibition of endonuclease activity by DPBA (**1**) and L-742,001 (**2**). Increasing concentrations of molecules (**1**) and (**2**) were incubated with 20 µ*M* protein and 1 µ*M* single-stranded RNA. The reaction products were analyzed in 20% polyacrylamide/8 *M* urea gels. The lane labelled NC lacked protein in the reaction mixture, while the lane labelled EDTA contained all reagents plus 5 m*M* EDTA.

In order to characterize the effect of the DKAs (**1**) and (**2**) on the endonuclease domain, we first investigated the thermal stability of ENDO-WT and its D88A and D118A mutants (ENDO-D88A and ENDO-D118A, respectively). The two ENDO mutations (D88A and D118A) were selected to elucidate the roles of the two aspartates in metal and ligand binding. D88A is a mutation of the key residue supposedly involved in bivalent metal-ion coordination, while D118A is a mutation of a conserved residue distant from the active site in the vicinity of the catalytic pocket (Morin *et al.*, 2010 ▸). To determine the thermal stability of ENDO-WT and its mutants, we used a thermofluorescence-based assay. In this assay, the fluorescence of a hydrophobic dye is measured as it binds to hydrophobic regions of the protein that are solvent-exposed as the protein is denatured by heating. The melting temperature (*T*_m_) of the protein is then determined from a temperature-dependent fluorescence curve. As shown in Fig. 2 ▸(*a*), the changes in the *T*_m_ of ENDO-WT and the ENDO-D118A mutant are similar, in contrast to those for the ENDO-D88A mutant.

In the absence of ions the *T*_m_ for both ENDO-WT and ENDO-D118A is around 40°C, whereas that for ENDO-D88A is 10°C higher, indicating that the enzyme is more stable. The addition of divalent ions (Mg^2+^, Mn^2+^ or a mixture of both) increases the *T*_m_ by ∼5°C for both ENDO-WT and ENDO-D118A. On the other hand, ENDO-D88A presents no significant increase in *T*_m_ under similar conditions. These results show the following. (i) ENDO-WT and ENDO-D118A are stabilized by divalent ions, but ENDO-D88A is not. (ii) Asp118 makes no direct or indirect interactions with the ions and has virtually no influence on the stability of the protein. This result was confirmed by our structural study (see §[Sec sec3.2]3.2). (iii) ENDO-D88A is not affected by either the presence or the absence of divalent ions, indicating that Asp88 is involved in divalent ion binding.

We next assessed the effect of di­valent metal ions and ligand binding on ENDO-WT and its mutants (Fig. 2 ▸ *a*). For both ENDO-WT and ENDO-D118A, the addition of either DPBA (**1**) or L-742,001 (**2**) in the presence of metal ions led to a significant positive *T*_m_ shift of greater than 10°C, which confirmed the high affinity of these ligands for the enzyme. No effect was observed when using ENDO-D88A. This latter result provides evidence that in absence of divalent ions the enzyme displays no affinity for the compounds. The increase in *T*_m_ indicates a gain in stability owing to the binding effect of the compounds to ENDO complexed with divalent ions. As no gain of stability was observed with the D88A mutant, a mutation that abrogates metal ion binding, we infer that the binding of ligands (**1**) and (**2**) is performed through metal-ion chelation.

#### 3.1.2. Binding affinity by MST and ITC   

To further characterize the ligand-binding mode, we used microscale thermophoresis to determine the affinity of ENDO-WT and the ENDO-D88A and ENDO-D118A mutants for DPBA (**1**) and L-742,001 (**2**) in the presence of divalent metal ions (Mg^2+^ and Mn^2+^). MST detects changes in the hydration shell of biomolecules and measures their inter­actions under close-to-native conditions. Any change in the hydration shell of proteins owing to changes in their structure affects the thermophoretic movement, which is used to determine binding affinities with high accuracy and sensitivity. The results summarized in Table 2 ▸ show that the affinities of ENDO-WT and ENDO-D118A for DPBA (**1**) are in the same (micromolar) range. However, in the case of L-742,001 (**2**) the *K*_d_ values for ENDO-WT and ENDO-D118A were about tenfold and 20-fold lower than that for DPBA (**1**), respectively. This suggests that both ENDO-WT and ENDO-D118A display a higher affinity for L-742,001 (**2**) than for DPBA (**1**).

Interestingly, for both compounds no significant affinity was recorded either for ENDO-WT without metal ions in the experimental buffer or for ENDO-D88A. This result corroborates the idea that the binding of the DKAs (**1**) and (**2**) takes place *via* the chelation of divalent cations and that Asp88 is key in this interaction.

We further validated the results obtained with DPBA (**1**) using isothermal titration calorimetry (ITC) as an orthogonal assay. The *K*_d_ of DPBA (**1**) for ENDO-WT and the two mutants ENDO-D118A and ENDO-D88A were determined and the data were of the same order as those determined by MST (Supplementary Fig. S2). ENDO-WT and ENDO-D118A displayed affinities in the low-micromolar range, while no interaction was detected for ENDO-D88A, confirming the earlier results.

### 3.2. ENDO-WT and ENDO-D118A holoenzyme structures   

In the original study of LCMV ENDO (Morin *et al.*, 2010 ▸), the crystallization conditions did not allow the formation of complexes with cofactors (ionic or otherwise). This was in part owing to the organization of the molecules in the crystal asymmetric unit, with one symmetric molecule occupying the catalytic site. Therefore, for this structural study to be successful it was critical to search for new crystallization conditions that would allow the molecules to form ionic complexes. The present ENDO-WT structure clearly shows the two Mg^2+^ ions directly coordinated by the catalytic Asp88 and, through a network of water molecules, by Glu101 and Lys114 (Supplementary Fig. S3*a*). From our observation of 100 crystals during the screening and analysis of work performed on the *Lassa virus* endonuclease domain, this coordination event seems to be rare. Indeed, in the structure of the *Lassa virus* endonuclease domain reported by Wallat *et al.* (2014 ▸) similar residues are involved, with a very dense network of water molecules stabilizing the ions. On the other hand, if we compare these results with the structure of the *Lassa virus* endonuclease domain reported by Reguera *et al.* (2016 ▸), we can see that the coordination of the two Mn^2+^ ions involved the corresponding residues Asp89, Glu102 and Lys115, a residue from the asymmetric unit (Glu3) and no waters. Together, these data indicate that the affinity of the *Arenaviridae* endonuclease for its catalytic ions is low and that the metal-ion binding at the catalytic site is the result of a random event in the absence of an RNA substrate.

In the ENDO-WT structure, the water molecules mimic the positions of the nonbridged O atoms of the RNA phosphodiester backbone involved in binding to the ions. This observation reminded us of previous comments on ribonuclease III, for which the substrate is proposed to bind to the enzyme independently and prior to the catalytic ions (Nicholson, 2014 ▸). Likewise, this is also the case for the *Arenaviridae* exonuclease mechanism, in which the RNA contributes to the formation of the binding site of the catalytic ion (Jiang *et al.*, 2013 ▸).

Therefore, we propose that in the case of the LCMV endonuclease domain the RNA substrate is a necessary contributor to the formation of a transitioning ion-binding/catalytic site that allows a two-metal-ion catalytic mechanism (TMIC).

In this study, we decided to assess the impact of a key residue within the catalytic site (Morin *et al.*, 2010 ▸) but *a priori* not involved in the mechanism. For this, we mutated a conserved aspartate to alanine (D118A). From a structural point of view, we confirmed by crystallography that the mutation has no effect on the overall LCMV endonuclease structure (Supplementary Fig. S3*b*), as preliminarily shown by the study of the thermal stability of ENDO-D118A by DSF.

### 3.3. DPBA–ENDO-WT complex   

**Figure 3:**
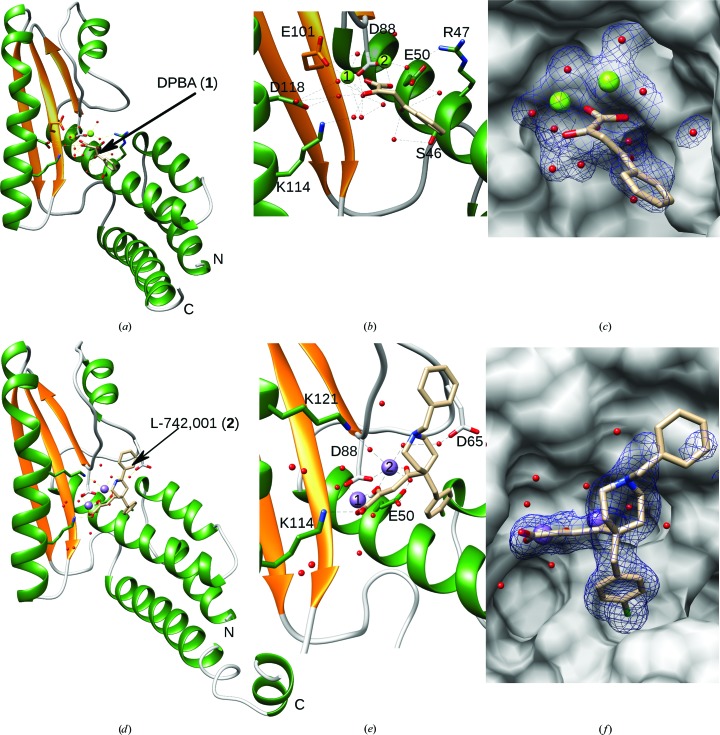
Crystal structures of the ENDO protein in complex with DPBA (**1**) and L-742,001 (**2**). LCMV endonuclease structures complexed with (**1**) (*a*, *b*, *c*) or (**2**) (*d*, *e*, *f*) are shown. Structures are represented as ribbons with helices in green and strands in gold, while the compound is represented as sticks. Mg^2+^ and Mn^2+^ ions are represented as light green and purple spheres, respectively. (*b*) and (*e*) show enlargements of the catalytic site showing the strong coordination of waters, ions and catalytic residues of the molecule. (*c*) and (*f*) show a 2*F*_o_ − *F*_c_ OMIT map corresponding to the compound (1σ).

To understand the molecular interactions between DPBA (**1**) and ENDO-WT, we solved the structure of their complex. The DPBA–ENDO-WT complex structure was determined at 1.88 Å resolution in space group *P*4_1_. DPBA (**1**) binds directly to the two Mg^2+^ ions in the active site, as clearly shown by the 2*F*_o_ − *F*_c_ OMIT map (Figs. 3 ▸ *a*, 3 ▸ *b* and 3 ▸ *c*). However, this binding mode is rather unusual compared with the binding configuration observed for DPBA (**1**) bound to the active sites of *Orthobunyavirus* endonuclease and the influenza PA N_ter_ (Reguera *et al.*, 2010 ▸; Kowalinski *et al.*, 2012 ▸; DuBois *et al.*, 2012 ▸). Indeed, DPBA (**1**) binds the first ion through the carboxylic group and the enolizable α-ketone and the second ion through both the α- and γ-ketones. In the DPBA–ENDO-WT structure the three adjacent and coplanar O atoms of the DKA motif of DPBA (**1**) bind the two metal ions as follows: Mg1 is coordinated by the carboxylic group and the enolizable α-ketone, whereas Mg2 is coordinated by the carboxyl group only (Figs. 3 ▸ *a* and 3 ▸ *b* and Supplementary Fig. S4*a*). The γ-ketone is flipped by about 180° and forms hydrogen bonds to two water molecules that mediate interaction with Ser46 and Glu50. The phenyl group of DPBA (**1**) makes no direct interactions with the residues in the active site.

This difference in binding mode might be owing to the enlarged nature of the active site of the LCMV ENDO *versus* *Orthobunyavirus* or influenza virus endonucleases, allowing the compound to adopt alternative conformations.

### 3.4. L-742,001–ENDO-WT complex   

The L-742,001–ENDO-WT complex was determined in the same space group as the DPBA–ENDO-WT complex, *P*4_1_, at 1.97 Å resolution. In contrast to DPBA (**1**), L-742,001 (**2**) adopts a conformation that is identical to that reported for influenza PA N_ter_ (Kowalinski *et al.*, 2012 ▸; DuBois *et al.*, 2012 ▸). The two metal ions are chelated by the three coplanar O atoms of L-742,001 (**2**) as indicated by the 2*F*_o_ − *F*_c_ OMIT map (Figs. 3 ▸ *d*, 3 ▸ *e* and 3 ▸ *f*, and Supplementary Fig. S4*b*). One of the Mn^2+^ ions is coordinated by Asp88 and Glu50 as well as Asp65 and Lys121 *via* bridging water molecules and by two O atoms of the ligand. The second metal ion is coordinated by Cys102, Asp88, two water molecules and two O atoms of the ligand, leading to an octahedral geometry for both ions. The O atom of the ligand also makes a hydrogen bond to Lys114, which is an important catalytic residue. The chlorobenzyl and benzyl­piperidine groups are oriented in opposite directions perpendicular to the dioxobutanoic acid. The chlorobenzyl group enters into a pocket comprising Arg47, Ser46, Lys43 and Glu50. The piperidine moiety, on the other hand, is poorly defined owing to weak electron density compared with the rest of the molecule. This suggests that some rotational flexibility exists owing to weak interactions between this part of the molecule and residues in the flexible region (residues 83–85) connecting the fourth α-helix to the first β-strand of the protein. Moreover, we observed an alternative conformation for the side chain of Asp88, suggesting that the presence of the compound removing the ion was sufficient to alter the ‘resting’ position of the side chain.

The overall three-structure comparison (holo structure and two ligand-complexed structures) raises an interesting observation concerning the way that ENDO coordinates the ions depending on the active-site environment. Indeed, in the holo structure the ions are coordinated by three residues, Asp88, Glu101 and Lys114, and in the DPBA-complexed structure the ions are also coordinated by three residues, Asp88, Glu101 and Asp118 (*via* a water molecule). On the other hand, in the L-742,001-complexed structure the ions are coordinated by Glu50, Asp88 and Leu121. The obvious difference between the two ligands lies in the fact that DPBA (**1**) makes no direct interaction with the enzyme, while L-742,001 (**2**) does interact through its chloro­benzyl group. This observation shows that any perturbation in the active site, such as a ligand, can trigger ion mobility. Figuratively, the ions bounce like a pinball in the active site to establish a dynamic new coordination.

### 3.5. Evaluation of DPBA (**1**) and L-742,001 (**2**) in an *in vitro* LCMV endonuclease-activity assay   

Having characterized the interactions between the two ligands and ENDO-WT, we proceeded to test the efficacy of compounds DPBA (**1**) and L-742,001 (**2**) in inhibiting the LCMV endonuclease activity in an *in vitro* endonuclease assay. A single-stranded 5′-radio-labelled RNA was incubated with ENDO-WT at a range of compound concentrations. Cleavage was analysed on a denaturing polyacrylamide gel and visualized on a phosphoimager (Fig. 2 ▸ *b*). As expected, DPBA (**1**) and L-742,001 (**2**) display inhibition of LCMV endonuclease activity; however, both compounds exhibit weak activity in the submillimolar range. DPBA (**1**) is slightly more active than L-742,001 (**2**), as its efficacy appears from 250 µ*M*, whereas that of L-742,001 (**2**) only appears from 1 m*M*. DPBA (**1**) and L-742,001 (**2**) are both relatively fair binders but very weak inhibitors of ENDO. However, both were described to be potent inhibitors of *Orthobunyavirus* and *Orthomyxovirus* endo­nuclease domains at micromolar and submicromolar levels. Their potency appeared to be relative to their mode of binding, which involves both ion chelation and hydrophobic or electrostatic interactions with some amino acids in the vicinity of the active site (Reguera *et al.*, 2010 ▸; Kowalinski *et al.*, 2012 ▸; DuBois *et al.*, 2012 ▸). ENDO accommodates DPBA (**1**) and L-742,001 (**2**); however, the binding mode is only driven and is predominately mediated by metal chelation. Indeed, if the ions are removed then the binding is completely lost (Fig. 2 ▸). The lack of polar inter­actions between the compound and the amino acids of the active site leads to residual flexibility and suboptimal efficacy and makes them ligands but weak inhibitors. Our assumption in the use of DKAs is that the compound competes with the RNA for the ions at the active site, thus creating a stable complex that acts as a steric inhibitor. To design potent inhibitors, a full-occupancy binding mode should be obtained. In order to maximize our chances, we re-explored *Arenaviridae* ENDO sequence–structure conservation to guide the design of new DKAs that are able to establish hydrophobic inter­actions with amino acids of the ENDO active site.

As its chemical synthesis is more accessible, we chose DPBA (**1**) to guide compound optimization, and it will be the starting point for structure-based drug development.

### 3.6. Compound optimization and *in silico* evaluation   

**Figure 4:**
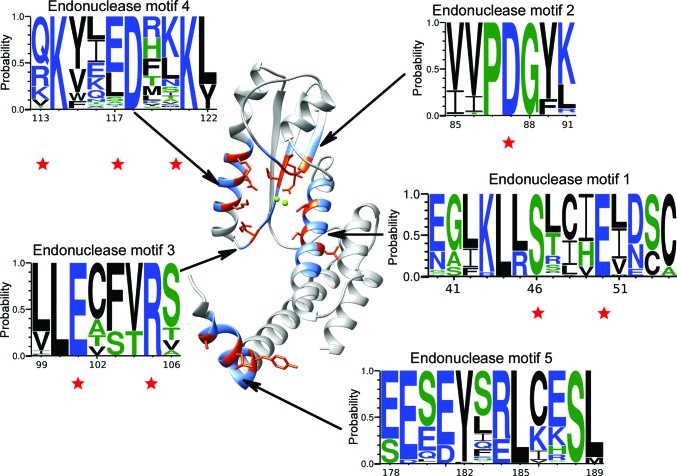
The five motifs (blue) that are conserved across all available sequences of the *Arenaviridae* endonuclease domain plotted on the LCMV structure (grey). Conserved motifs are shown in *WebLogo* representation. The size of the letter is representative of the frequency with which it observed in the alignment (with a probability of 1 being identity). The numbering of the residues refers to the sequence of LCMV. Motifs 1–4 represent the four positions that can be used to anchor a ligand over all *Arenaviridae*. Residues marked with a star correspond to key residues involved in the active site or in substrate binding, and their side chains are highlighted on the structure (orange).

From a sequence point of view, *Arenaviridae* endonuclease has only five absolutely conserved regions, from which five motifs can be derived. Four of the five motifs (motifs 1–4) are located in the vicinity of the catalytic site (Fig. 4 ▸). The central motif 2, PDG, is the endonuclease catalytic signature. Motifs 1, 3 and 4 surround motif 2, forming a hydrophobic slit below the catalytic site. Motif 1 is the furthest away but contributes together with the end of motif 2 to the formation of one side of the slit owing to their hydrophobic nature. Motifs 3 and 4 form the other side of the slit. They are both hydrophobic but are punctuated with conserved polar amino acids including Glu101, Lys114, Asp118 and Lys121. These motifs show that despite years of evolution and viral diversity across the family, these residues are invariant and consequently highly important for function or fold integrity. Therefore, for compound optimization these motifs can be used as solid ground to anchor compatible chemical moieties that would reinforce the binding and increase the inhibitory effect. In this spirit, and after gaining insights into how DPBA (**1**) interacts with ENDO, we decided to explore the effect of modifications of the phenyl ring of DPBA (**1**) in an attempt to improve its interactions with the amino acids of the active site and consequently its binding efficacy.

We hypothesized that increasing the aromatic content of the ligands should reinforce the binding of the corresponding DKAs by establishing hydrophobic contacts with the residues of motifs 3 and 4 (Fig. 4 ▸).

**Figure 5:**
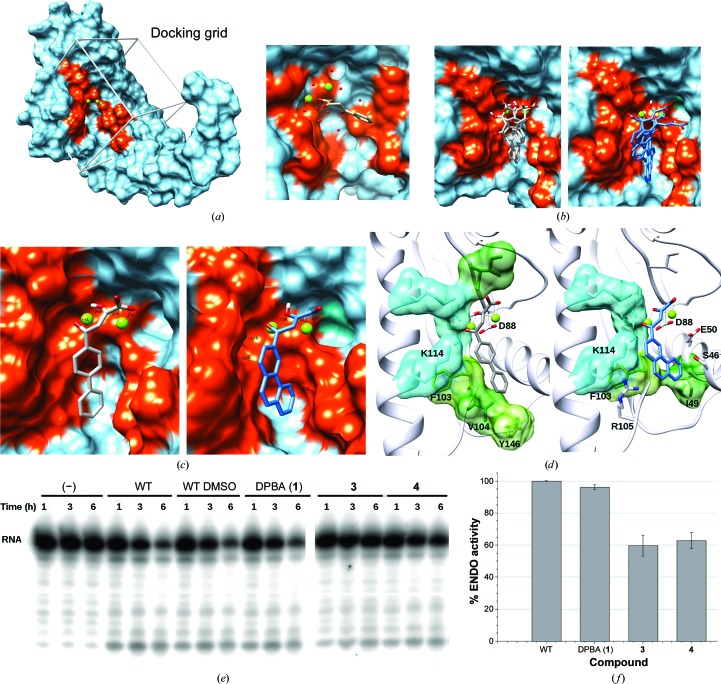
(*a*) Structure of ENDO-WT complexed with Mg ions and the docking grid. The surface is coloured cyan and the conserved motif is coloured orange. Mg^2+^ ions are represented as light green spheres. The position of DPBA (**1**) is shown in the right panel. (*b*) Selection of the best docking poses of compounds (**3**) and (**4**). (*c*) Enlargement of the best pose on the cavity surface. (*d*) Enlargement of the best pose with the residues involved in binding. These are coloured according to hydrophobicity from cyan (least) to green (most). (*e*) Polyacrylamide/8 *M* urea gels of compounds (**3**) and (**4**) (50 µ*M*) incubated with 20 µ*M* protein and 1 µ*M* single-stranded RNA. (*f*) Quantification of endonuclease activity in the presence of DMSO and compounds (**1**), (**3**) and (**4**).

We designed two new DKAs, compounds (**3**) and (**4**), withbiphenyl and phenanthryl moieties, respectively (Fig. 1 ▸ *b*). The selected aromatic rings reinforce the rigidity and hydrophobicity of the compound while increasing its electronegativity, potentially allowing broader contacts in pockets. The biphenyl structure allows a larger degree of freedom compared with the phenanthryl structure, allowing the second ring to rotate. Docking simulations confirmed our hypothesis, showing that increasing the number and type of aromatic components within the ligand [molecules (**3**) and (**4**)] provides a second ‘anchorage’ into the targeted conserved area of the catalytic pocket (Figs. 5 ▸ *a*, 5 ▸ *b* and 5 ▸ *c*). The poses of the molecules have a binding energy (*E*) ranging between −7.2 and −5.5 kcal mol^−1^. This interaction can be described as follows: (i) the DKA moiety interacts with the first metal ion through the carboxylic group and the enolizable α-ketone and the second metal ion through both the α- and γ-ketones, and (ii) the aromatic rings of compounds (**3**) and (**4**) generate hydrophobic interactions of the conserved region of motifs 3 and 4 with residues Phe103, Val104, Lys114 and Tyr146, and of motifs 1, 3 and 4 with residues Ile49, Glu50, Cys102, Phe103, Val104, Arg105 and Lys114, respectively (Fig. 5 ▸ *d* and Supplementary Fig. S5).

With this perspective, we synthesized two new DKA compounds (**3**) and (**4**). These compounds were first evaluated for their affinity for ENDO-WT by MST; DKAs (**3**) and (**4**) displayed *K*_d_ values of 0.05 ± 0.02 and 0.25 ± 0.07 µ*M*, respectively. Both new DKAs showed a tenfold to 100-fold better affinity compared with DPBA (**1**), indicating that the modifications provide additional interactions with ENDO-WT leading to better binding. We also tested the inhibition of DKAs (**3**) and (**4**) using our *in vitro* endonuclease-activity assay (Figs. 5 ▸ *e* and 5 ▸ *f*). We observed that the intensity of the undigested RNA in the presence of the DKAs (**3**) and (**4**) remains stronger compared with DPBA (**1**) (Fig. 5 ▸ *e*). Quantification of the intensity of the bands reflects a diminution of enzyme activity in the presence of the new molecules (**3**) and (**4**). This is an indication that compound modifications increasing hydrophobic interactions lead to a gain in potency (conservatively estimated at about 35%). These findings are a proof of concept that the design of optimized inhibitors, despite being challenging, is possible when proper structural information is available.

## 4. Conclusions   

We report four crystallographic structures of the LCMV endonuclease domain: a complex with two divalent ions (Mg^2+^), the D118A mutant and the first two structures of complexes with ligands – the DKAs DPBA (**1**) and L-742,001 (**2**). These structures provide the first detailed structural information on a specific ligand bound to an arenavirus endonuclease. Our data show that the presence of ions in the holo structure is in fact a rare event. The structures show that the metal-ion binding depends on the number of water molecules that are able to create a mesh of interaction around the ions. This suggests that the catalytic ions responsible for the two-metal-ion catalysis reaction are most likely brought in by the RNA substrate itself. This study shows that Asp88 is the critical residue that mediates metal-ion binding. Moreover, based on an extensive sequence analysis of all *Arenaviridae* endonuclease domains, we have identified four motifs that can be used as anchors for the specificity of optimized compounds targeting this domain. Our preliminary results, based on two new optimized DKAs, suggest that aromatic ring modifications improve binding and mostly anti-ENDO activity. In spite of the challenge of an open active site, this study is a proof of concept that knowledge of ligand-bound structures can guide the design of optimized inhibitors against the LCMV endonuclease domain.

## Supplementary Material

PDB reference: LCMV endonuclease domain, complex with Mg^2+^ ions, 5ltf

PDB reference: complex with DPBA, 5ltn

PDB reference: complex with L-742,001, 5t2t

PDB reference: D118A mutant, 5lts

Supplementary Figures and Tables.. DOI: 10.1107/S2052252518001021/lz5019sup1.pdf

## Figures and Tables

**Table 1:** Data-collection and refinement statistics for the LCMV endonuclease domain complexed with ions and compounds and for its D118A mutant Values in parentheses are for the highest resolution shell.

	LCMV ENDO complexed with Mg^2+^ ions	LCMV ENDO complexed with DPBA (**1**)	LCMV ENDO complexed with L-742,001 (**2**)	LCMV ENDO-D118A mutant
PDB code	5ltf	5ltn	5t2t	5lts
Beamline	ID23-1, ESRF	PROXIMA-1, SOLEIL	ID23-2, ESRF	PROXIMA-1, SOLEIL
Wavelength (Å)	0.976	0.978	0.873	0.978
Resolution range (Å)	48.46–2.43 (2.52–2.43)	53.59–1.88 (1.95–1.88)	54.06–1.97 (2.04–1.97)	53.57–2.51 (2.60–2.51)
Space group	*P*4_1_	*P*4_1_	*P*4_1_	*C*222_1_
*a*, *b*, *c* (Å)	108.4, 108.4, 54.1	107.2, 107.2, 53.8	108.1, 108.1, 54.1	144.8, 159.2, 52.7
α, β, γ (°)	90, 90, 90	90, 90, 90	90, 90, 90	90, 90, 90
Molecules in asymmetric unit	2	2	2	2
Total reflections	67793 (6444)	241187 (14092)	308124 (31957)	73597 (7143)
Unique reflections	23573 (2252)	49896 (4927)	44686 (4457)	21183 (2063)
Multiplicity	2.9 (2.9)	4.8 (2.9)	6.9 (7.2)	3.5 (3.4)
Completeness (%)	96.0 (96.0)	99.6 (99.5)	100 (100)	99.0 (100)
Mean *I*/σ(*I*)	13.61 (1.58)	8.54 (1.20)	12.51 (2.02)	14.17 (2.09)
Wilson *B* factor (Å^2^)	57.12	37.53	33.03	58.27
*R*_merge_	0.046 (0.607)	0.094 (0.868)	0.094 (0.870)	0.060 (0.594)
*R*_meas_	0.056 (0.744)	0.105 (1.15)	0.102 (0.938)	0.071 (0.704)
CC_1/2_	0.999 (0.586)	0.996 (0.416)	0.998 (0.715)	0.997 (0.649)
No. of reflections used in refinement	22999	49844	44682	21091
No. of free reflections	1932	2457	2210	1004
*R*_work_	0.18 (0.27)	0.19 (0.29)	0.18 (0.27)	0.20 (0.27)
*R*_free_	0.23 (0.33)	0.21 (0.29)	0.22 (0.30)	0.22 (0.29)
No. of atoms in structure				
Total	3240	3353	3586	3184
Macromolecules	3080	3064	3193	3063
Ligands	2	60	47	8
R.m.s.d., bonds (Å)	0.009	0.010	0.008	0.013
R.m.s.d., angles (°)	1.09	1.10	1.86	1.67
Ramachandran plot				
Favoured (%)	92	97	97	97
Allowed (%)	5.7	1.9	2.3	2.7
Outliers† (%)	2.3	1.1	0.7	0.3
Average *B* factor (Å^2^)	75.92	50.30	41.98	74.32

† Outliers are in highly flexible regions of the protein.

**Table 2:** Determination of the dissociation constants of ENDO proteins (WT and D118A and D88A mutants) with DPBA (**1**) and L-742,001 (**2**) in the presence or absence of ions by microscale thermophoresis (MST) The concentration of labelled protein was kept constant at 100 n*M*, while the concentration of the compound was varied from 500 µ*M* to 15 n*M* to yield 16 serial concentrations. ND, not detectable.

	*K*_d_ (µ*M*)					
	WT		D118A		D88A	
Compounds	Ion	No ion	Ion	No ion	Ion	No ion
DPBA (**1**)	5.38 ± 2.06	ND	18.76 ± 3.88	ND	ND	ND
L-742,001 (**2**)	0.51 ± 0.11	ND	0.89 ± 0.06	ND	ND	ND

## Appendix A Chemistry

All reagents and anhydrous solvents were purchased from Aldrich and were used without further drying. All reactions involving air-sensitive or moisture-sensitive compounds were performed under an argon atmosphere using oven-dried glassware and syringes. NMR spectra were determined in DMSO-d_6_ on a Jeol spectrometer (400 MHz). Chemical shifts were expressed in p.p.m. and coupling constants (*J*) are given in Hertz (s, singlet; d, doublet; m, multiplet). HPLC/MS spectra were recorded on a Thermo Fisher Accela 600 (detection 260 nm) with an Hypersil-Gold C18 (2.5 µm, 2.1 × 50 mm) column and a Finnigan Surveyor MSQ (MS) under electrospray ionization (ESI) with positive and negative ionization-mode detection. Compounds were eluted with a gradient of a solution of 5–100% 0.01% formic acid in acetonitrile solution with a 500 µl min^−1^ flow rate.

### A1. General procedure for the preparation of compounds (**3**) and (**4**)

Sodium (20 mmol) was added slowly to cold methanol in an ice–water bath to give a 2 *M* solution of sodium methoxide. The appropriate aryl methyl ketone (5 mmol) and dimethyl oxalate (10 mmol) were dissolved in dry THF/DME [1:1(*v*:*v*)] and the solution was added dropwise to the freshly prepared sodium methoxide. The mixture was stirred at room temperature for 8 h. The resulting solid was filtered off, washed with cold methanol and diethyl ether and dried. The sodium ketoenolate ester obtained was dissolved in water and stirred at room temperature for 1 h. The solution was then acidified to pH 3–4 by adding 1 *N* HCl and kept at 4°C for 2 h. The precipitate was filtered off, washed with cold water and dried. The ketoenol ester obtained was dissolved in THF/MeOH [1:1(*v*:*v*)] and a solution of 2 *N* NaOH was added dropwise. The resulting clear solution was stirred at room temperature for 1 h. The mixture was acidified to pH 2 by adding 1 *N* HCl and the precipitate formed was filtered off, washed with cold water and dried under vacuum. The crude material obtained was purified by crystallizing it from diethyl ether, which yielded the desired aryl diketo acids (**3**) and (**4**).

### A2. 2-Hydroxy-4-(biphenyl-4-yl)-4-oxobut-2-enoic acid (**3**)

This was obtained as a yellow solid (212 mg, 27% global yield) using 4-acetylbiphenyl as the starting material. Tr-HPLC/MS, 3.87 min. LC-MS (ESI^−^), 266.97 [*M*–H]^−^; HRMS ESI^−^ calculated for C_16_H_12_O_4_ [*M*–H]^−^, 267.0663; found 267.0663. ^1^H NMR (400 MHz, DMSO-d_6_) δ (p.p.m.): 8.16 (d, 2H, *J* = 8.1 Hz), 7.88 (d, 2H, *J* = 8.1 Hz), 7.77 (d, 2H, *J* = 7.8 Hz), 7.54–7.50 (m, 2H), 7.47–7.38 (m, 1H), 7.15 (s, 1H), 4.60 (s, 2H, methylenic proton of the diketo tautomer, ketoenol/diketo ratio = 0.1). ^13^C NMR (100 MHz, DMSO-d_6_) δ (p.p.m.): 189.73, 170.28, 163.16, 145.33, 138.61, 133.39, 129.14, 128.63, 127.24, 127.03, 97.87.

### A3. 2-Hydroxy-4-oxo-4-(phenanthren-3-yl)but-2-enoic acid (**4**)

This was obtained as a yellow solid (185 mg, 23% global yield) using 3-acetylphenanthrene as the starting material. Tr-HPLC/MS, 4.10 min. LC-MS (ESI^−^), 290.94 [*M*–H]^−^; HRMS ESI^−^ calculated for C_18_H_12_O_4_ [*M*–H]^−^, 291.0663; found, 291.0664. ^1^H NMR (400 MHz, DMSO-d_6_) δ (p.p.m.): 9.37 (s, 1H), 9.01 (s, 1H), 8.16–7.90 (m, 5H), 7.76–7.70 (m, 2H), 7.00 (s, 1H), 4.74 (s, 2H, methylenic proton of the diketo tautomer, ketoenol/diketo ratio = 0.15). ^13^C NMR (100 MHz, DMSO-d_6_) δ (p.p.m.): 187.61, 174.57, 164.17, 134.01, 131.79, 131.79, 129.97, 129.37, 129.04, 128.72, 127.46, 127.37, 126.32, 124.89, 123.28, 122.52, 99.13.
