# Supplementary material for: Crystal structures of Lymphocytic choriomeningitis virus endonuclease domain complexed with diketo-acid ligands
Source: IUCrJ. 2018 Feb 22;5(Pt 2):223–35. doi: 10.1107/S2052252518001021 (PMC5947727; doi:10.1107/S2052252518001021)
Supplement: Supplementary file 1 [file m-05-00223-sup1.pdf]

IUCrJ

Volume 5 (2018)

Supporting information for article:

Crystal structures of *Lymphocytic choriomeningitis virus* endonuclease domain complexed with diketo acid ligands

Magali Saez-Ayala, Elsie Laban Yekwa, Mauro Carcelli, Bruno Canard, Karine Alvarez and François Ferron

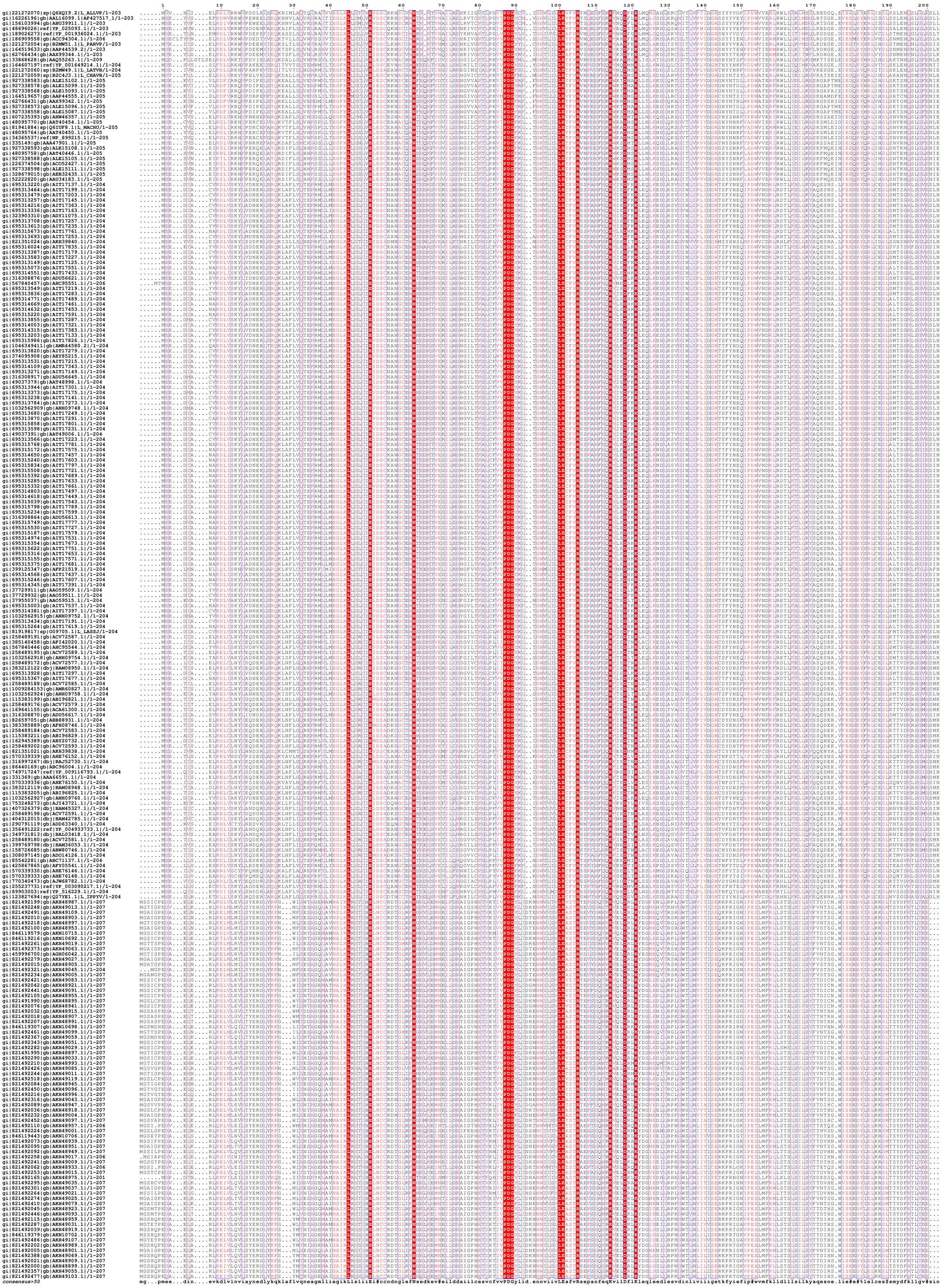

Figure S1 Alignment of 245 sequences of *Arenaviridae* L ENDO domain prepared with ESPript.

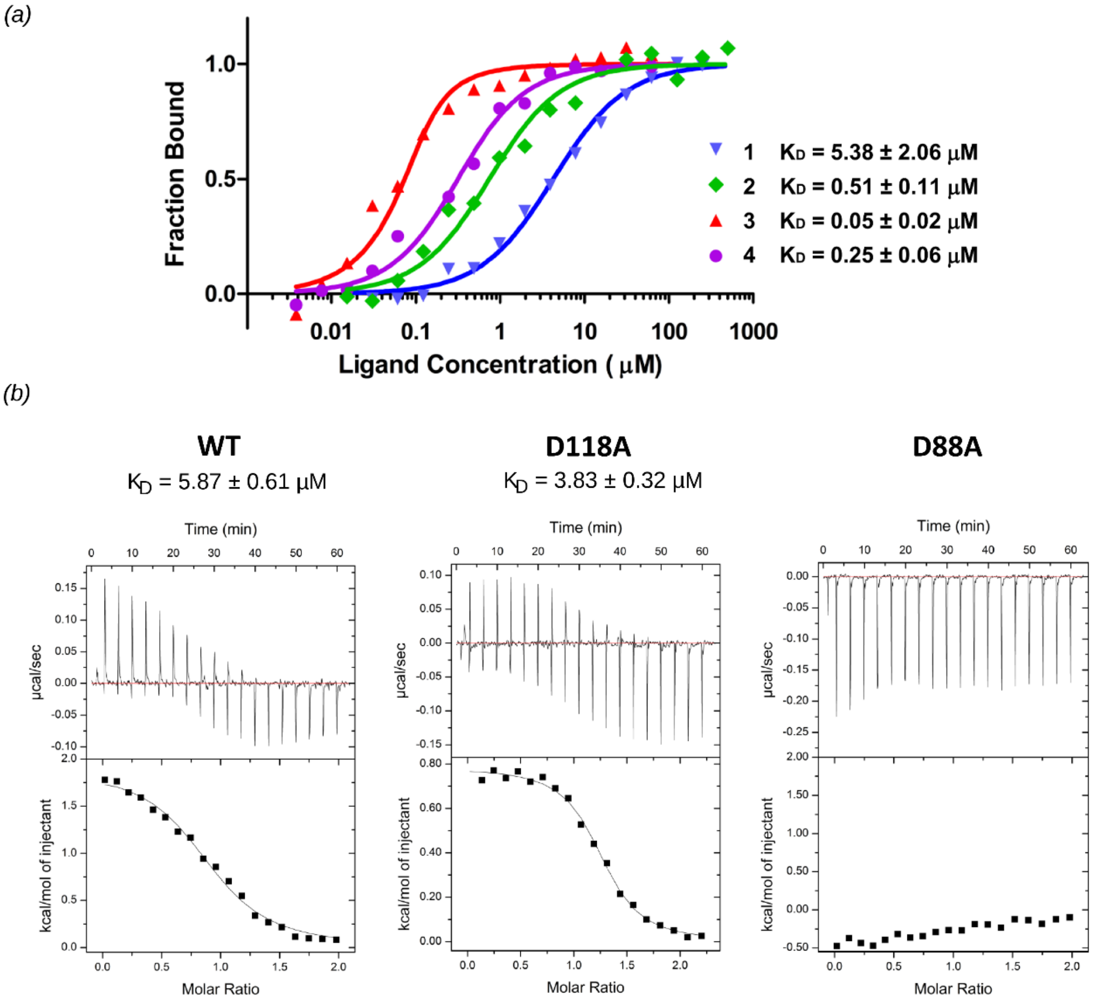

Figure S2 *(a)* Representative binding curves for ENDO-WT to DPBA (**1**), L-742,001 (**2**), **3** and **4,** determined by microscale thermophoresis (MST). Fraction Bound represents the baseline corrected normalized fluorescence for each compound with normalization to 1 for amplitude. *(b)* Representative ITC titrations for ENDO proteins with DPBA (**1**) at 25 °C as a function of the molar ratio of ligand to protein. The upper panel shows the ITC raw data and the lower panel presents the integrated heats of each injection. Binding isotherms were fitted to the raw data using one-site binding model to determine the K_D.

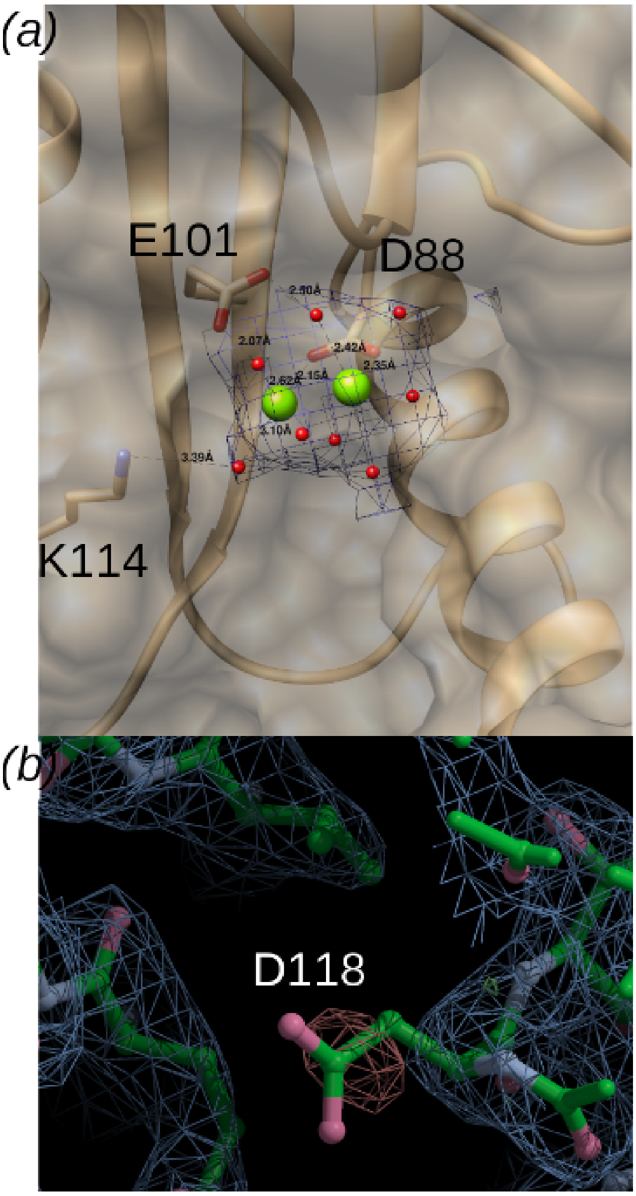

Figure S3 (a) Zoom of the catalytic site of the holo-enzyme structure of the LCMV endonuclease with the 2 catalytic Mg^{2+} coordinated by D88 and a network of water molecules. (b) 2Fo-Fc Map of the D118A mutant. Overall structure of the endonuclease LCMV in not affected by this mutation.

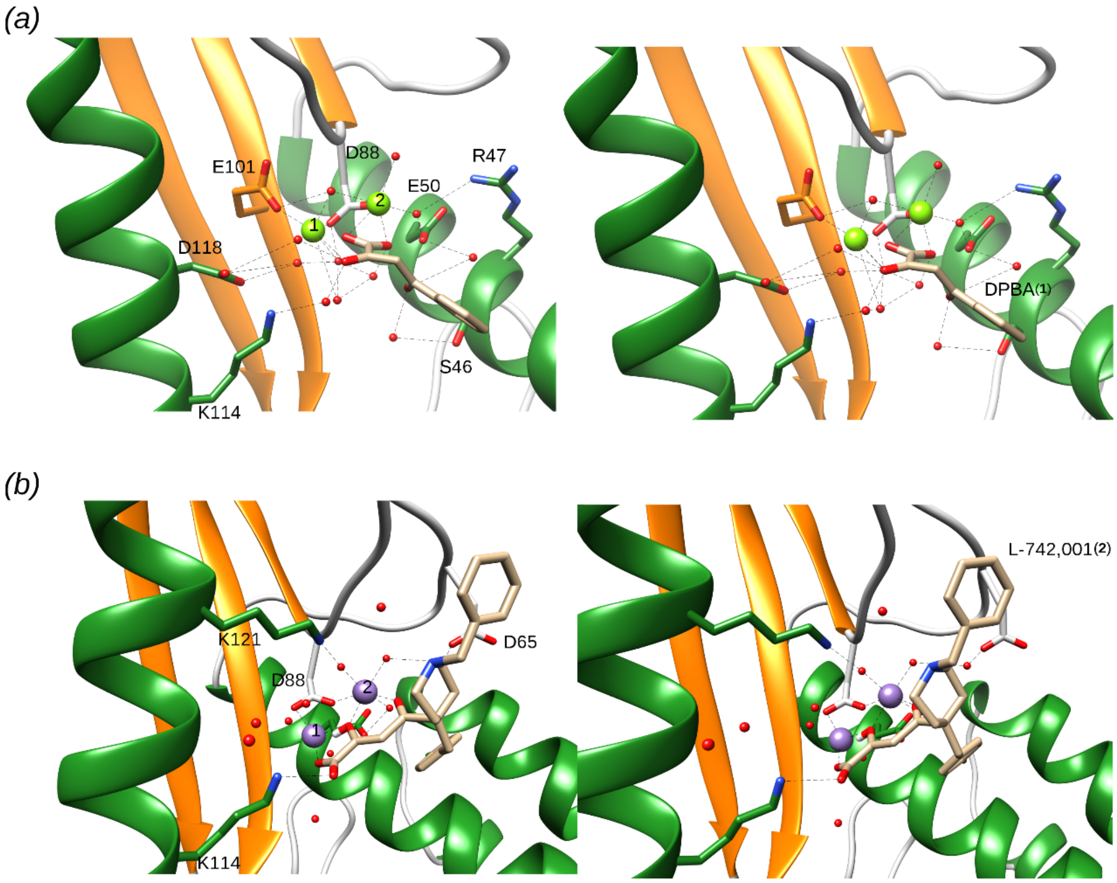

Figure S4 Zoom of the catalytic site showing the intense water ions molecule catalytic residues coordination in stereo view. Crystal structures of ENDO protein in complex with (a) DPBA (**1**) and (b) L-742,001 (**2**). Structures are represented as ribbon with helices in green and strands in gold while the compound is represented in sticks. Mg^{2+} and Mn^{2+} ions are represented as light green and purple spheres respectively.

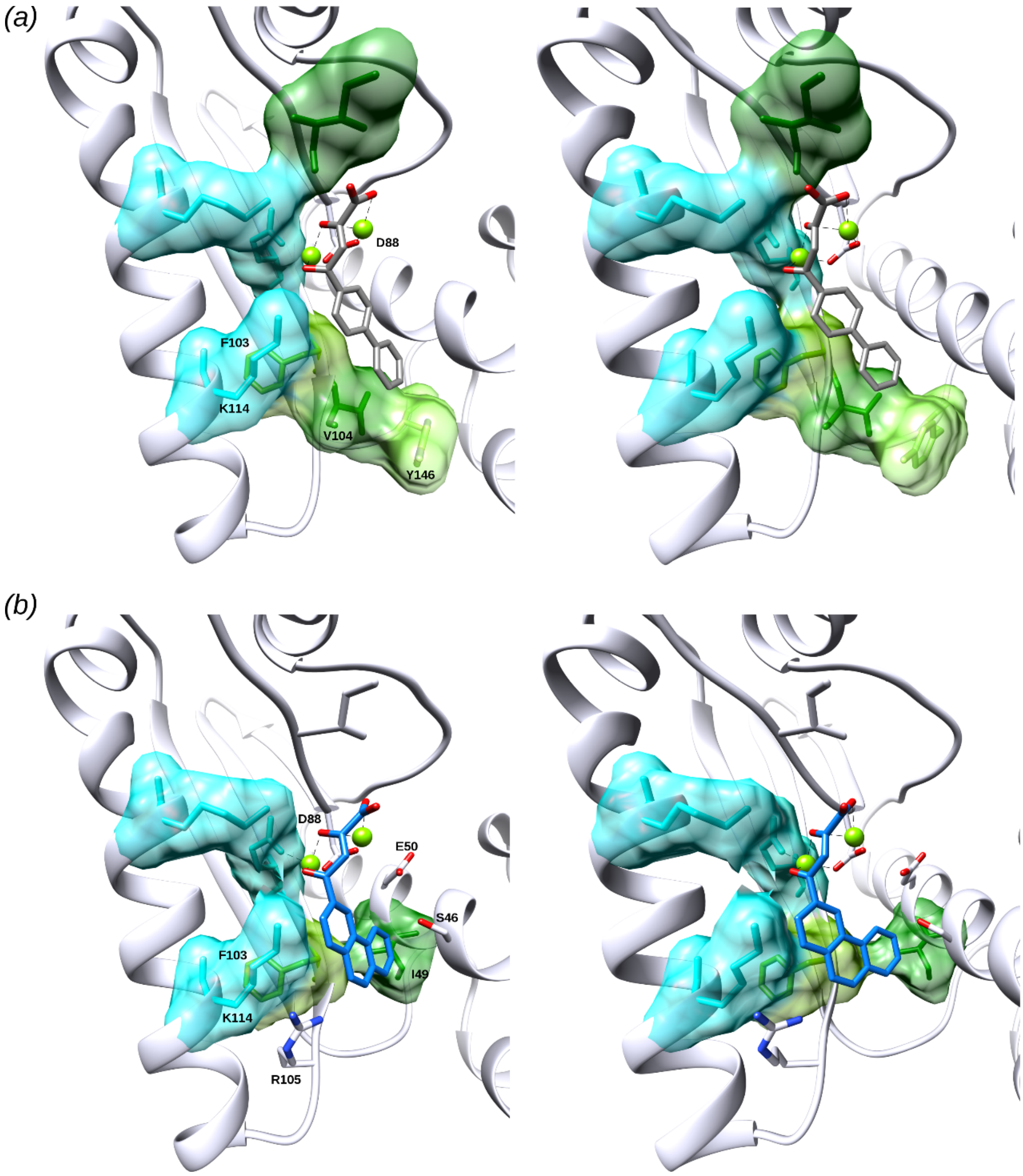

Figure S5 Zoom of best pose of molecules **3** (a) and **4** (b) in stereo view. Residues and their surface involved in binding are coloured according to hydrophobicity from cyan (the least) to green (the most). Structures are represented as ribbon while the compound is represented in sticks. Mg^{2+} ions are represented as light green spheres.

Table S1 Arenaviridae Endonuclease domain L NCBI reference number.

Supplemementary Table1 : Liste of L protein sequences used in bioinformatic analysis.	
**N°**	**NCBI GI Number**
1	gi|221272070|sp|Q6XQI9.2|L_ALLVP/1-203 RecName: Full=RNA-directed RNA polymerase L
2	gi|156103994|gb|ABU39911.1|/1-203 L polymerase [Pichinde mammarenavirus]
3	gi|16226196|gb|AAL16099.1|AF427517_1/1-203 L polymerase [Pichinde mammarenavirus]
4	gi|48696026|ref|YP_025093.1|/1-203 L protein [Pirital mammarenavirus]
5	gi|186909558|gb|ACC94304.1|/1-206 L protein [Tamiami mammarenavirus]
6	gi|189026273|ref|YP_001936024.1|/1-203 L protein [Flexal mammarenavirus]
7	gi|221272054|sp|B2MW51.1|L_PARVP/1-203 RecName: Full=RNA-directed RNA polymerase L
8	gi|62766434|gb|AAX99344.1|/1-203 RNA-dependent RNA polymerase [Bear Canyon mammarenavirus]
9	gi|33868628|gb|AAQ55263.1|/1-209 L protein [Sabia mammarenavirus]
10	gi|164519642|gb|AAP44542.2|/1-209 L protein [Sabia mammarenavirus]
11	gi|164607197|ref|YP_001649214.1|/1-204 L protein [Oliveros mammarenavirus]
12	gi|62766431|gb|AAX99342.1|/1-205 RNA-dependent RNA polymerase [Amapari mammarenavirus]
13	gi|48095770|gb|AAT40454.1|/1-205 L protein [Machupo mammarenavirus]
14	gi|221272059|sp|B2C4J3.1|L_CHAVB/1-205 RecName: Full=RNA-directed RNA polymerase L
15	gi|81941884|sp|Q6IUF8.1|L_MACHU/1-205 RecName: Full=RNA-directed RNA polymerase L
16	gi|48095764|gb|AAT40450.1|/1-205 L protein [Machupo mammarenavirus]
17	gi|34365537|ref|NP_899215.1|/1-205 L protein [Machupo mammarenavirus]
18	gi|221272060|sp|B2MW49.1|L_LATVB/1-204 RecName: Full=RNA-directed RNA polymerase L
19	gi|927338583|gb|ALE15102.1|/1-205 RNA-directed RNA polymerase L [Guanarito mammarenavirus]
20	gi|927338568|gb|ALE15093.1|/1-205 RNA-directed RNA polymerase L [Guanarito mammarenavirus]
21	gi|927338578|gb|ALE15099.1|/1-205 RNA-directed RNA polymerase L [Guanarito mammarenavirus]
22	gi|927338573|gb|ALE15096.1|/1-205 RNA-directed RNA polymerase L [Guanarito mammarenavirus]
23	gi|927338558|gb|ALE15087.1|/1-205 RNA-directed RNA polymerase L [Guanarito mammarenavirus]
24	gi|607235393|gb|AHW46357.1|/1-205 RNA-dependent RNA polymerase [Tacaribe mammarenavirus]
25	gi|335149|gb|AAA47901.1|/1-205 L protein [Tacaribe mammarenavirus]
26	gi|164519657|gb|AAP44555.2|/1-205 L protein [Cupixi mammarenavirus]
27	gi|695315673|gb|AIT17761.1|/1-204 polymerase [Lassa mammarenavirus]
28	gi|927338588|gb|ALE15105.1|/1-205 RNA-directed RNA polymerase L [Junin mammarenavirus]
29	gi|695315073|gb|AIT17551.1|/1-204 polymerase [Lassa mammarenavirus]
30	gi|316308864|gb|ADU56613.1|/1-204 polymerase [Lassa mammarenavirus]
31	gi|695315039|gb|AIT17543.1|/1-204 polymerase [Lassa mammarenavirus]
32	gi|695315003|gb|AIT17537.1|/1-204 polymerase [Lassa mammarenavirus]
33	gi|695314650|gb|AIT17457.1|/1-204 polymerase [Lassa mammarenavirus]
34	gi|927338593|gb|ALE15108.1|/1-205 RNA-directed RNA polymerase L [Junin mammarenavirus]
35	gi|695315187|gb|AIT17579.1|/1-204 polymerase [Lassa mammarenavirus]
36	gi|695315234|gb|AIT17599.1|/1-204 polymerase [Lassa mammarenavirus]
37	gi|695315172|gb|AIT17575.1|/1-204 polymerase [Lassa mammarenavirus]
38	gi|695314803|gb|AIT17497.1|/1-204 polymerase [Lassa mammarenavirus]
39	gi|695315332|gb|AIT17661.1|/1-204 polymerase [Lassa mammarenavirus]
40	gi|695314618|gb|AIT17449.1|/1-204 polymerase [Lassa mammarenavirus]
41	gi|695315240|gb|AIT17603.1|/1-204 polymerase [Lassa mammarenavirus]
42	gi|695315834|gb|AIT17797.1|/1-204 polymerase [Lassa mammarenavirus]
43	gi|695315768|gb|AIT17781.1|/1-204 polymerase [Lassa mammarenavirus]
44	gi|695315508|gb|AIT17721.1|/1-204 polymerase [Lassa mammarenavirus]
45	gi|695315285|gb|AIT17633.1|/1-204 polymerase [Lassa mammarenavirus]
46	gi|695315392|gb|AIT17689.1|/1-204 polymerase [Lassa mammarenavirus]
47	gi|695315530|gb|AIT17727.1|/1-204 polymerase [Lassa mammarenavirus]
48	gi|695315432|gb|AIT17703.1|/1-204 polymerase [Lassa mammarenavirus]
49	gi|48095758|gb|AAT40446.1|/1-205 L protein [Junin mammarenavirus]
50	gi|695315252|gb|AIT17611.1|/1-204 polymerase [Lassa mammarenavirus]
51	gi|927338598|gb|ALE15111.1|/1-205 RNA-directed RNA polymerase L [Junin mammarenavirus]
52	gi|226374504|gb|ACO52427.1|/1-205 L protein [Junin mammarenavirus]
53	gi|695315246|gb|AIT17607.1|/1-204 polymerase [Lassa mammarenavirus]
54	gi|695315354|gb|AIT17673.1|/1-204 polymerase [Lassa mammarenavirus]
55	gi|695315622|gb|AIT17751.1|/1-204 polymerase [Lassa mammarenavirus]
56	gi|695315749|gb|AIT17777.1|/1-204 polymerase [Lassa mammarenavirus]
57	gi|695316024|gb|AIT17835.1|/1-204 polymerase [Lassa mammarenavirus]
58	gi|695315316|gb|AIT17653.1|/1-204 polymerase [Lassa mammarenavirus]
59	gi|695315798|gb|AIT17789.1|/1-204 polymerase [Lassa mammarenavirus]
60	gi|316308876|gb|ADU56621.1|/1-204 polymerase [Lassa mammarenavirus]
61	gi|695314551|gb|AIT17433.1|/1-204 polymerase [Lassa mammarenavirus]
62	gi|695315375|gb|AIT17681.1|/1-204 polymerase [Lassa mammarenavirus]
63	gi|695314632|gb|AIT17453.1|/1-204 polymerase [Lassa mammarenavirus]
64	gi|695314771|gb|AIT17489.1|/1-204 polymerase [Lassa mammarenavirus]
65	gi|695315155|gb|AIT17571.1|/1-204 polymerase [Lassa mammarenavirus]
66	gi|695314974|gb|AIT17531.1|/1-204 polymerase [Lassa mammarenavirus]
67	gi|328679015|gb|AEB32435.1|/1-205 L protein [Junin mammarenavirus]
68	gi|52222820|gb|AAU34183.1|/1-205 L protein [Junin mammarenavirus]
69	gi|695313836|gb|AIT17283.1|/1-204 polymerase [Lassa mammarenavirus]
70	gi|399125347|gb|AFP21519.1|/1-204 large RNA-dependent RNA polymerase [Lujo mammarenavirus]
71	gi|695313220|gb|AIT17137.1|/1-204 polymerase [Lassa mammarenavirus]

72	gi|695313464|gb|AIT17199.1|/1-204 polymerase [Lassa mammarenavirus]
73	gi|695314669|gb|AIT17461.1|/1-204 polymerase [Lassa mammarenavirus]
74	gi|567840457|gb|AHC95551.1|/1-206 L polymerase [Lassa mammarenavirus]
75	gi|695314216|gb|AIT17363.1|/1-204 polymerase [Lassa mammarenavirus]
76	gi|695313257|gb|AIT17145.1|/1-204 polymerase [Lassa mammarenavirus]
77	gi|323903310|gb|ADY11075.1|/1-204 L protein [Lassa mammarenavirus]
78	gi|695313238|gb|AIT17141.1|/1-204 polymerase [Lassa mammarenavirus]
79	gi|695313479|gb|AIT17203.1|/1-204 polymerase [Lassa mammarenavirus]
80	gi|695313708|gb|AIT17257.1|/1-204 polymerase [Lassa mammarenavirus]
81	gi|695313693|gb|AIT17253.1|/1-204 polymerase [Lassa mammarenavirus]
82	gi|695313784|gb|AIT17273.1|/1-204 polymerase [Lassa mammarenavirus]
83	gi|695315367|gb|AIT17677.1|/1-204 polymerase [Lassa mammarenavirus]
84	gi|695313336|gb|AIT17163.1|/1-204 polymerase [Lassa mammarenavirus]
85	gi|695314568|gb|AIT17437.1|/1-204 polymerase [Lassa mammarenavirus]
86	gi|695313598|gb|AIT17231.1|/1-204 polymerase [Lassa mammarenavirus]
87	gi|695315858|gb|AIT17801.1|/1-204 polymerase [Lassa mammarenavirus]
88	gi|49037391|gb|AAT49006.1|/1-204 L protein [Lassa mammarenavirus]
89	gi|695315986|gb|AIT17826.1|/1-204 polymerase [Lassa mammarenavirus]
90	gi|695313203|gb|AIT17133.1|/1-204 polymerase [Lassa mammarenavirus]
91	gi|1046349411|gb|AMR44580.2|/1-204 L polymerase [Lassa mammarenavirus]
92	gi|695313271|gb|AIT17149.1|/1-204 polymerase [Lassa mammarenavirus]
93	gi|49037379|gb|AAT48998.1|/1-204 L protein [Lassa mammarenavirus]
94	gi|695313549|gb|AIT17219.1|/1-204 polymerase [Lassa mammarenavirus]
95	gi|695313361|gb|AIT17171.1|/1-204 polymerase [Lassa mammarenavirus]
96	gi|695313531|gb|AIT17215.1|/1-204 polymerase [Lassa mammarenavirus]
97	gi|695313149|gb|AIT17125.1|/1-204 polymerase [Lassa mammarenavirus]
98	gi|695314109|gb|AIT17343.1|/1-204 polymerase [Lassa mammarenavirus]
99	gi|374095908|gb|AEY85215.1|/1-204 L protein [Lassa mammarenavirus]
100	gi|81919817|sp|O09705.1|L_LASSJ/1-204 RecName: Full=RNA-directed RNA polymerase L
101	gi|316308870|gb|ADU56617.1|/1-204 polymerase [Lassa mammarenavirus]
102	gi|695313387|gb|AIT17179.1|/1-204 polymerase [Lassa mammarenavirus]
103	gi|316308917|gb|ADU56645.1|/1-204 polymerase [Lassa mammarenavirus]
104	gi|695313583|gb|AIT17227.1|/1-204 polymerase [Lassa mammarenavirus]
105	gi|695314003|gb|AIT17321.1|/1-204 polymerase [Lassa mammarenavirus]
106	gi|695314315|gb|AIT17383.1|/1-204 polymerase [Lassa mammarenavirus]
107	gi|695313855|gb|AIT17287.1|/1-204 polymerase [Lassa mammarenavirus]
108	gi|695313434|gb|AIT17191.1|/1-204 polymerase [Lassa mammarenavirus]
109	gi|695313820|gb|AIT17279.1|/1-204 polymerase [Lassa mammarenavirus]
110	gi|695315264|gb|AIT17619.1|/1-204 polymerase [Lassa mammarenavirus]
111	gi|258489191|gb|ACV72587.1|/1-204 L protein [Lymphocytic choriomeningitis mammarenavirus]
112	gi|115383199|gb|ABI96821.1|/1-204 L protein [Lymphocytic choriomeningitis mammarenavirus]
113	gi|695313680|gb|AIT17249.1|/1-204 polymerase [Lassa mammarenavirus]
114	gi|695313870|gb|AIT17291.1|/1-204 polymerase [Lassa mammarenavirus]
115	gi|1032562909|gb|ANH09748.1|/1-204 polymerase [Lassa mammarenavirus]
116	gi|37729911|gb|AAO59509.1|/1-204 polymerase [Lassa mammarenavirus]
117	gi|695313566|gb|AIT17223.1|/1-204 polymerase [Lassa mammarenavirus]
118	gi|383212122|dbj|BAM08950.1|/1-204 L protein [Lymphocytic choriomeningitis mammarenavirus]
119	gi|695313928|gb|AIT17297.1|/1-204 polymerase [Lassa mammarenavirus]
120	gi|37729932|gb|AAO59511.1|/1-204 polymerase [Lassa mammarenavirus]
121	gi|695314345|gb|AIT17391.1|/1-204 polymerase [Lassa mammarenavirus]
122	gi|695313944|gb|AIT17301.1|/1-204 polymerase [Lassa mammarenavirus]
123	gi|695313373|gb|AIT17175.1|/1-204 polymerase [Lassa mammarenavirus]
124	gi|695314381|gb|AIT17397.1|/1-204 polymerase [Lassa mammarenavirus]
125	gi|1032562915|gb|ANH09752.1|/1-204 polymerase [Lassa mammarenavirus]
126	gi|115383205|gb|ABI96825.1|/1-204 L protein [Lymphocytic choriomeningitis mammarenavirus]
127	gi|82659705|gb|ABB88931.1|/1-204 L protein [Lymphocytic choriomeningitis mammarenavirus]
128	gi|383385889|gb|AFH08746.1|/1-204 polymerase [Lymphocytic choriomeningitis mammarenavirus]
129	gi|821351024|gb|AKH39840.1|/1-204 RNA-dependent RNA polymerase [Okahandja virus]
130	gi|567840446|gb|AHC95544.1|/1-204 L polymerase [Lassa mammarenavirus]
131	gi|258489184|gb|ACV72583.1|/1-204 L protein [Lymphocytic choriomeningitis mammarenavirus]
132	gi|162945389|gb|ABY20732.1|/1-204 RNA-dependent RNA polymerase [Dandenong virus]
133	gi|331369|gb|AAA66591.1|/1-204 L protein [Lymphocytic choriomeningitis mammarenavirus]
134	gi|158726685|gb|ABW80746.1|/1-204 L protein [Lymphocytic choriomeningitis mammarenavirus]
135	gi|385140458|gb|AFI42020.1|/1-204 large RNA-dependent RNA polymerase protein [Lymphocytic choriomeningitis mammarenavirus]
136	gi|258489195|gb|ACV72589.1|/1-204 L protein [Lymphocytic choriomeningitis mammarenavirus]
137	gi|749717247|ref|YP_009116793.1|/1-204 polymerase [Gairo virus]
138	gi|86440169|gb|ABC96004.1|/1-204 L protein [Lymphocytic choriomeningitis mammarenavirus]
139	gi|258489188|gb|ACV72585.1|/1-204 L protein [Lymphocytic choriomeningitis mammarenavirus]
140	gi|1009284153|gb|AMR60827.1|/1-204 RNA-dependent RNA polymerase [Lymphocytic choriomeningitis mammarenavirus]
141	gi|753248273|gb|AJI43721.1|/1-204 polymerase [Praomys arenavirus]
142	gi|290791119|gb|ADD63340.1|/1-204 L [Merino Walk mammarenavirus]
143	gi|316997267|dbj|BAJ52730.1|/1-204 L protein [Lymphocytic choriomeningitis mammarenavirus]
144	gi|1032562918|gb|ANH09754.1|/1-204 polymerase [Lassa mammarenavirus]
145	gi|258489172|gb|ACV72577.1|/1-204 L protein [Lymphocytic choriomeningitis mammarenavirus]
146	gi|169641155|gb|ACA61300.1|/1-204 L protein [Lymphocytic choriomeningitis mammarenavirus]
147	gi|115383211|gb|ABI96829.1|/1-204 L protein [Lymphocytic choriomeningitis mammarenavirus]
148	gi|37955037|gb|AAO59515.1|/1-204 polymerase [Lassa mammarenavirus]
149	gi|1032562924|gb|ANH09758.1|/1-204 polymerase [Lassa mammarenavirus]
150	gi|85542281|gb|ABC71137.1|/1-204 L protein [Mopeia mammarenavirus]

151	gi|308097145|gb|ADO14126.1|/1-204 L protein [Lymphocytic choriomeningitis mammarenavirus]
152	gi|356491222|ref|YP_004933733.1|/1-204 L gene product [Luna mammarenavirus]
153	gi|407326379|dbj|BAM45327.1|/1-204 L protein [Luna mammarenavirus]
154	gi|570339339|gb|AHE76152.1|/1-204 RNA-dependent RNA polymerase [Loie River virus]
155	gi|258489202|gb|ACV72593.1|/1-204 L protein [Lymphocytic choriomeningitis mammarenavirus]
156	gi|570339336|gb|AHE76150.1|/1-204 RNA-dependent RNA polymerase [Loie River virus]
157	gi|404312015|dbj|BAM42785.1|/1-204 RNA-dependent RNA polymerase [Luna mammarenavirus]
158	gi|349731813|dbj|BAL03418.1|/1-204 RNA-dependent RNA polymerase [Luna mammarenavirus]
159	gi|1032562927|gb|ANH09760.1|/1-204 polymerase [Lassa mammarenavirus]
160	gi|770340473|gb|AJW68782.1|/1-204 L protein [Rat arenavirus 1]
161	gi|821351021|gb|AKH39838.1|/1-204 RNA-dependent RNA polymerase [Mariental virus]
162	gi|383212119|dbj|BAM08948.1|/1-204 L protein [Lymphocytic choriomeningitis mammarenavirus]
163	gi|258489198|gb|ACV72591.1|/1-204 L protein [Lymphocytic choriomeningitis mammarenavirus]
164	gi|425867865|gb|AFY05541.1|/1-204 polymerase RDRP [Mopeia Lassa virus reassortant 29]
165	gi|258489176|gb|ACV72579.1|/1-204 L protein [Lymphocytic choriomeningitis mammarenavirus]
166	gi|399769798|dbj|BAM36053.1|/1-204 L protein [Luna mammarenavirus]
167	gi|258489180|gb|ACV72581.1|/1-204 L protein [Lymphocytic choriomeningitis mammarenavirus]
168	gi|570339333|gb|AHE76148.1|/1-204 RNA-dependent RNA polymerase [Cardamones virus]
169	gi|255237731|ref|YP_003090217.1|/1-204 polymerase [Morogoro virus]
170	gi|570339330|gb|AHE76146.1|/1-204 RNA-dependent RNA polymerase [Cardamones virus]
171	gi|89903003|ref|YP_516229.1|/1-204 L protein [Mobala mammarenavirus]
172	gi|123827694|sp|Q27YE1.1|L_IPPYV/1-204 RecName: Full=RNA-directed RNA polymerase L
173	gi|821492491|gb|AKH49109.1|/1-207 L polymerase [unidentified Reptarenavirus]
174	gi|821492261|gb|AKH49019.1|/1-207 L polymerase [unidentified Reptarenavirus]
175	gi|459996700|gb|AGH06042.1|/1-207 L polymerase [ROUT virus]
176	gi|821492218|gb|AKH48997.1|/1-207 L polymerase [unidentified Reptarenavirus]
177	gi|821492224|gb|AKH49001.1|/1-207 L polymerase [unidentified Reptarenavirus]
178	gi|821492010|gb|AKH48903.1|/1-207 L polymerase [unidentified Reptarenavirus]
179	gi|821492100|gb|AKH48953.1|/1-207 L polymerase [unidentified Reptarenavirus]
180	gi|821492015|gb|AKH48905.1|/1-207 L polymerase [unidentified Reptarenavirus]
181	gi|821492321|gb|AKH49045.1|/1-204 L polymerase [unidentified Reptarenavirus]
182	gi|821492461|gb|AKH49099.1|/1-207 L polymerase [unidentified Reptarenavirus]
183	gi|821492279|gb|AKH49027.1|/1-207 L polymerase [unidentified Reptarenavirus]
184	gi|821492234|gb|AKH49005.1|/1-207 L polymerase [unidentified Reptarenavirus]
185	gi|821492248|gb|AKH49013.1|/1-207 L polymerase [unidentified Reptarenavirus]
186	gi|846119579|gb|AKN10715.1|/1-207 RdRp [Aurora borealis virus]
187	gi|821492199|gb|AKH48987.1|/1-207 L polymerase [unidentified Reptarenavirus]
188	gi|821492373|gb|AKH49063.1|/1-207 L polymerase [unidentified Reptarenavirus]
189	gi|846119307|gb|AKN10698.1|/1-207 RdRp [Suri Vanera virus]
190	gi|821492367|gb|AKH49059.1|/1-207 L polymerase [unidentified Reptarenavirus]
191	gi|821492032|gb|AKH48915.1|/1-207 L polymerase [unidentified Reptarenavirus]
192	gi|821492207|gb|AKH48991.1|/1-207 L polymerase [unidentified Reptarenavirus]
193	gi|821492216|gb|AKH48996.1|/1-207 L polymerase [unidentified Reptarenavirus]
194	gi|821492018|gb|AKH48907.1|/1-207 L polymerase [unidentified Reptarenavirus]
195	gi|846119216|gb|AKN10692.1|/1-207 RdRp [Aurora borealis virus]
196	gi|821492282|gb|AKH49029.1|/1-207 L polymerase [unidentified Reptarenavirus]
197	gi|821492343|gb|AKH49051.1|/1-207 L polymerase [unidentified Reptarenavirus]
198	gi|821492295|gb|AKH49035.1|/1-207 L polymerase [unidentified Reptarenavirus]
199	gi|821492441|gb|AKH49091.1|/1-207 L polymerase [unidentified Reptarenavirus]
200	gi|821492421|gb|AKH49083.1|/1-207 L polymerase [unidentified Reptarenavirus]
201	gi|821492165|gb|AKH48975.1|/1-201 L polymerase [unidentified Reptarenavirus]
202	gi|821492076|gb|AKH48941.1|/1-207 L polymerase [unidentified Reptarenavirus]
203	gi|821492450|gb|AKH49096.1|/1-207 L polymerase [unidentified Reptarenavirus]
204	gi|821492105|gb|AKH48955.1|/1-207 L polymerase [unidentified Reptarenavirus]
205	gi|821492042|gb|AKH48921.1|/1-207 L polymerase [unidentified Reptarenavirus]
206	gi|821492290|gb|AKH49033.1|/1-207 L polymerase [unidentified Reptarenavirus]
207	gi|821492426|gb|AKH49085.1|/1-207 L polymerase [unidentified Reptarenavirus]
208	gi|821491990|gb|AKH48895.1|/1-207 L polymerase [unidentified Reptarenavirus]
209	gi|821492210|gb|AKH48993.1|/1-207 L polymerase [unidentified Reptarenavirus]
210	gi|821492110|gb|AKH48957.1|/1-206 L polymerase [unidentified Reptarenavirus]
211	gi|821492258|gb|AKH49017.1|/1-206 L polymerase [unidentified Reptarenavirus]
212	gi|821492244|gb|AKH49011.1|/1-207 L polymerase [unidentified Reptarenavirus]
213	gi|821492446|gb|AKH49093.1|/1-207 L polymerase [unidentified Reptarenavirus]
214	gi|821492241|gb|AKH49009.1|/1-207 L polymerase [unidentified Reptarenavirus]
215	gi|821491995|gb|AKH48897.1|/1-207 L polymerase [unidentified Reptarenavirus]
216	gi|821492062|gb|AKH48933.1|/1-206 L polymerase [unidentified Reptarenavirus]
217	gi|821492518|gb|AKH49119.1|/1-207 L polymerase [unidentified Reptarenavirus]
218	gi|821492084|gb|AKH48945.1|/1-207 L polymerase [unidentified Reptarenavirus]
219	gi|821492253|gb|AKH49015.1|/1-207 L polymerase [unidentified Reptarenavirus]
220	gi|821492316|gb|AKH49043.1|/1-207 L polymerase [unidentified Reptarenavirus]
221	gi|821492036|gb|AKH48918.1|/1-207 L polymerase [unidentified Reptarenavirus]
222	gi|821492232|gb|AKH49004.1|/1-207 L polymerase [unidentified Reptarenavirus]
223	gi|821492045|gb|AKH48923.1|/1-207 L polymerase [unidentified Reptarenavirus]
224	gi|821492089|gb|AKH48947.1|/1-207 L polymerase [unidentified Reptarenavirus]
225	gi|821492095|gb|AKH48951.1|/1-207 L polymerase [unidentified Reptarenavirus]
226	gi|821492115|gb|AKH48959.1|/1-207 L polymerase [unidentified Reptarenavirus]
227	gi|821492452|gb|AKH49097.1|/1-207 L polymerase [unidentified Reptarenavirus]
228	gi|821492092|gb|AKH48949.1|/1-207 L polymerase [unidentified Reptarenavirus]
229	gi|821492021|gb|AKH48909.1|/1-207 L polymerase [unidentified Reptarenavirus]

230	gi|821492264|gb|AKH49021.1|/1-207 L polymerase [unidentified Reptarenavirus]
231	gi|821492410|gb|AKH49079.1|/1-207 L polymerase [unidentified Reptarenavirus]
232	gi|821492274|gb|AKH49025.1|/1-207 L polymerase [unidentified Reptarenavirus]
233	gi|846119443|gb|AKN10706.1|/1-207 RdRp [Hans Kompis virus]
234	gi|821492073|gb|AKH48939.1|/1-207 L polymerase [unidentified Reptarenavirus]
235	gi|821492388|gb|AKH49069.1|/1-207 L polymerase [unidentified Reptarenavirus]
236	gi|821492000|gb|AKH48899.1|/1-207 L polymerase [unidentified Reptarenavirus]
237	gi|846119379|gb|AKN10702.1|/1-207 RdRp [Tavallinen suomalainen mies virus]
238	gi|821492039|gb|AKH48919.1|/1-207 L polymerase [unidentified Reptarenavirus]
239	gi|821492202|gb|AKH48989.1|/1-207 L polymerase [unidentified Reptarenavirus]
240	gi|821492477|gb|AKH49103.1|/1-207 L polymerase [unidentified Reptarenavirus]
241	gi|821492357|gb|AKH49055.1|/1-207 L polymerase [unidentified Reptarenavirus]
242	gi|821492301|gb|AKH49037.1|/1-207 L polymerase [unidentified Reptarenavirus]
243	gi|821492005|gb|AKH48901.1|/1-207 L polymerase [unidentified Reptarenavirus]
244	gi|821492486|gb|AKH49107.1|/1-207 L polymerase [unidentified Reptarenavirus]
245	gi|821492287|gb|AKH49031.1|/1-207 L polymerase [unidentified Reptarenavirus]
